# Cybersicherheit von Gehirn-Computer-Schnittstellen

**DOI:** 10.1365/s43439-022-00046-x

**Published:** 2022-03-17

**Authors:** Mario Martini, Carolin Kemper

**Affiliations:** 1 Deutsche Universität für Verwaltungswissenschaften (DUV), Speyer, Deutschland; 2 Deutsches Forschungsinstitut für öffentliche Verwaltung (FÖV), Speyer, Deutschland

**Keywords:** IT-Sicherheit, Medizinprodukte, Wearables, Datensicherheit, Neurotechnologie, IT security, Medical devices, Wearables, Data security, Neurotechnology

## Abstract

Gehirn-Computer-Schnittstellen beflügeln die Hoffnung auf übermenschliche Kräfte: Sie versetzen Nutzer in die Lage, Prothesen und sonstige Geräte allein mit ihren Gedanken zu steuern. Je weiter die Entwicklung der neuen Technologie voranschreitet und in marktfähige Produkte mündet, desto sichtbarer rücken auch potenzielle Sicherheitsrisiken in den Fokus. Denn Angriffe auf Gehirn-Computer-Schnittstellen können neurologische Daten erspähen oder Gehirnaktivitäten manipulieren und dadurch verheerende Schäden verursachen. Der Beitrag geht der Frage auf den Grund, wie die Rechtsordnung den Risiken eines Angriffs auf Gehirn-Computer-Schnittstellen bislang begegnet – und wie sie ihnen künftig begegnen sollte.

## Abstract (en)

Brain-computer interfaces inspire visions of superhuman powers, enabling users to control protheses and other devices solely with their thoughts. But the rapid development and commercialization of this technology also brings security risks. Attacks on brain-computer interfaces may cause harrowing consequences for users, from eavesdropping on neurological data to manipulating brain activity. At present, data protection law, the regulation of medical devices, and the new rules on the sale of goods with digital elements all govern aspects of cybersecurity. There are, nevertheless, significant gaps. The article analyzes how the legal system currently addresses the risks of cyberattacks on brain–computer interfaces—and how policymakers could address such risks in the future.

## Gehirn-Computer-Schnittstellen auf dem Weg vom Medizinlabor in die Anwendungspraxis

Im Jahr 2077 könnten Gehirn-Computer-Schnittstellen^1^ so allgegenwärtig sein wie heute Smartphones: Wer seine kognitiven Fähigkeiten erweitern, Computersysteme mit Gedanken ansteuern und Prothesen intuitiv wie eigene Körperteile bewegen möchte, nimmt die Hilfe von „Cyberware“ in Anspruch. Dieses dystopisch anmutende Szenario malt jedenfalls das Computerspiel *Cyberpunk 2077* an die Leinwand der Zukunft:^2^ Als Bewohner der Metropole *Night City* kann sich jeder mit einem Neuroimplantat im Frontalkortex und einem Cyberdeck ausstatten.^3^ Die Spieler sind dadurch in der Lage, sog. „Quickhacks“ auszuführen: Diese infiltrieren gegnerische Netzwerke, um alle Personen und Geräte ausfindig machen, die damit verbunden sind. Das Arsenal der neuronalen Angriffe geht so weit, einem Gegner aus der Distanz Stromschläge zu versetzen, die Steuerung seiner Prothesen zu deaktivieren oder ihn gar zum „Selbstmord“ zu treiben.^4^

So futuristisch sich die virtuellen Welten in Cyberpunk 2077 auch präsentieren: Hinter der Technologie der Gehirn-Computer-Schnittstellen steht mehr als nur Science-Fiction. Bislang kommen sie primär als Medizinprodukte oder in der Forschung zum Einsatz – insbesondere um körperlich eingeschränkten Menschen (z. B. Paraplegiker)^5^ durch Sprachcomputer oder Prothesen [1, S. 112 ff., 189 ff.]^6^ Rehabilitationsleistungen zu erschließen. Unterdessen strömen auch die ersten Verbraucherprodukte auf den Markt.^7^ Als Teil der „Quantified Self“-Bewegung^8^ sind bspw. Kopfhörer verfügbar, die den Stress- oder Konzentrationslevel messen.^9^ EEG-basierte Schnittstellen ermöglichen es auch, Computerspiele zu steuern;^10^ pro futuro können sie gar die Spielerfahrung den emotionalen Reaktionen des Spielers anpassen [11, 12].

Es finden sich auch Schnittstellen, die Arbeitnehmer im Bereich der Arbeitssicherheit^11^ und des Gesundheitsschutzes („Workplace Wellness, Safety & Productivity“^12^) unterstützen sollen. Optisch lassen sich die Geräte kaum von einem handelsüblichen Headset unterscheiden. Es entwickelt sich ein Milliardenmarkt mit erheblichem Wachstumspotenzial [15].

Neuere Varianten von Schnittstellen können nicht nur die neurologischen Signale der Nutzer auswerten, sondern auch gezielt Gehirnnerven stimulieren.^13^ Zudem lassen sich die individuellen Gehirnaktivitätsmuster jedes Menschen – wie Fingerabdrücke – als Authentifizierungsinstrument einsetzen [17]. Auch interaktive Kunstprojekte, wie dasjenige des Künstlers *Yehuda Duenyas*, integrieren die neue Technik in ihr kreatives Instrumentarium: „The Ascent“ ermöglicht es Personen, in einem Klettergurt zu schweben und sich – basierend auf ihren Gehirnsignalen – auf und ab zu bewegen [18].

Die Forschung bringt derweil immer beeindruckendere Entwicklungen der Neurotechnologie hervor. So hat anlässlich der Fußball-WM 2014 in Brasilien ein Querschnittsgelähmter via Gehirn-Computer-Schnittstelle ein Exoskelett gesteuert und damit symbolisch den Anstoß ausgeführt [19]. Auch Menschen mit einer Querschnittslähmung, die alle vier Gliedmaßen betrifft (Tetraplegiker), treten unterdessen mit Gehirn-Computer-Schnittstellen im virtuellen „Cybathlon“ gegeneinander an [20]. Besonders medienwirksam hat *Elon Musk* mit seinem Unternehmen *Neuralink* der Welt demonstriert, was alsbald Realität sein könnte: Es lässt Affen mithilfe ihrer Neuroimplantate „MindPong“ spielen – mit dem erklärten Ziel, das Modell einer Fernsteuerung durch Denkleistung auf den Einsatz am Menschen zu übertragen [21].

Schenkt man der Trendforschung Glauben, gilt für Gehirn-Computer-Schnittstellen: „The sky is the limit“. Künftig könnten Astronauten mit ihrer Hilfe Roboterarme bei Reparaturen verwenden, ohne einen Außenbordeinsatz durchführen zu müssen.^14^ Auch Smart-Home-Systeme oder gar humanoide Roboter lassen sich in Zukunft womöglich mit dem Gehirn steuern [1, S. 245 ff.]: Dann reicht ein Gedankenbefehl, damit der Hausroboter eine Pizza in den Ofen schiebt und im Arbeitszimmer serviert.

Das Ende der Fahnenstange ist aber auch damit noch nicht erreicht: Technik-Auguren spekulieren unterdessen darauf, dass Gehirn-Computer-Schnittstellen als Teil eines *Cognitive Enhancement* eine Art Mensch 2.0 hervorbringen oder die nächsten Generationen gar zu Cyborgs [23, S. 492 ff.; 24, S. 828 f.] mutieren lassen könnten: Eine Schnittstelle kann die menschlichen kognitiven Fähigkeiten in bislang unbekannte Höhen schrauben, z. B. das Gedächtnis optimieren oder Informationen direkt aus dem Internet als globaler Wissensbibliothek herauslesen und in „Denkprozesse“ einspeisen [1, S. 262 f.]. Transhumanisten träumen sogar davon, dass Menschen dank Neuroimplantaten mit Computern verschmelzen.^15^ Neuroimplantate koppeln sich dann mit einem KI-System, um rechenintensive Aufgaben auszulagern [25, S. 72 f.; 26, S. 192]. Ebenso scheint die Vision am Horizont auf, dass Nanoroboter biologische neuronale Netze durch synthetische ersetzen und dadurch neue Verbindungen und Netzwerke bilden, welche drahtlos mit anderen (Gehirnen) kommunizieren könnten.^16^ Die Grenze zwischen technisch Möglichem und einer Extrapolation falscher Annahmen über den menschlichen Geist verschwimmt dabei aber zuweilen [30, S. 32].

### Erscheinungsformen von Gehirn-Computer-Schnittstellen

#### Technische Funktionsweise

So vielseitig die Funktionen und Einsatzgebiete der Technologie auch sind, so sehr folgen Gehirn-Computer-Schnittstellen einem einfachen technischen Bauprinzip: Sie verbinden das Gehirn mit einem Computer, z. B. mit einer mikroprozessorgesteuerten Prothese.^17^ Das zwischengeschaltete Kommunikationssystem erkennt die elektrophysiologischen Signale des Gehirns und übersetzt sie in Handlungsbefehle.

Da die Gehirnaktivitäten jedes Menschen individuell sind, muss das System die Signalmuster des Einzelnen in einem ersten Schritt lesen lernen.^18^ Seine Kernkomponente bildet daher die Software, die Gehirnsignale verarbeitet und dekodiert. Hinzu treten Apps, die z. B. neurologische Daten anzeigen und analysieren oder ihrem Nutzer Spiele anbieten.^19^

Die Handlungsmuster und -richtungen der Schnittstellen lassen sich im Grundsatz drei Gruppen zuordnen: passiven (a), aktiven (b) und stimulierenden (c) Schnittstellen.

##### *a) Passive Schnittstellen*

Passive Gehirn-Computer-Schnittstellen beschränken sich darauf, Gehirnaktivitäten zu messen,^20^ um sie anschließend einem Verhalten, mentalen Zustand oder der kognitiven Bewältigung einer Aufgabe zuzuordnen [2, S. 782].^21^ Sie stehen vor der technischen Herkulesaufgabe, die Gehirnaktivitäten zu dekodieren und zu klassifizieren. Das System korreliert dafür gemessene neuronale Signalmuster mit einer Aktivität (z. B. dem Heben des linken Arms) oder mit einem neuronalen Zustand (bspw. Anzeichen eines epileptischen Anfalls, Stress oder Depressionen).^22^

##### *b) Aktive Schnittstellen*

Aktive Gehirn-Computer-Schnittstellen können Gehirnaktivitäten nicht nur analysieren, sondern auch eine Aktion in der Außenwelt auslösen. Sie sind etwa dazu in der Lage, eine Prothese am Körper einer Person zu bewegen oder eine Mitteilung durch einen Sprachcomputer auszugeben [37]. Dafür muss der Nutzer ein spezifisches Gehirnaktivitätsmuster kognitiv (durch „Denken“) herbeiführen. Die aktive Schnittstelle dekodiert dieses (wie eine passive Gehirn-Computer-Schnittstelle) und löst darauf aufbauend den gewünschten Vorgang (z. B. eine Armbewegung) aus.

##### *c) Stimulierende Schnittstellen*

Anders als aktive erzeugen simulierende Gehirn-Computer-Schnittstellen elektrische Impulse „nach innen“, um bestimmte Gehirnareale – und dadurch spezifische Gehirnaktivitäten – zu beeinflussen, etwa mit dem Ziel, Muskelzittern (Tremores) bei der Parkinson-Krankheit oder Anfällen bei Epilepsiepatienten vorzubeugen [1, S. 210 ff.; 2, S. 806 ff.; 38, v. a. S. 361 f.].^23^ Ähnlich wie aktive Gehirn-Computer-Schnittstellen induzieren auch hier spezifische neurologische Signalmuster diese Impulse. Ein Hauptanwendungsfall ist die sog. *Deep Brain Stimulation*: Sie versetzt dem Gehirn – ähnlich wie ein Herzschrittmacher – elektrische Impulse und lindert dadurch die Symptome mancher Krankheiten, wie Parkinson, Epilepsie oder Depressionen [1, S. 216 f.; 40].^24^

*Bidirektionale* Gehirn-Computer-Schnittstellen gehen noch einen Schritt weiter, indem sie die Fähigkeiten aktiver und stimulierender Gehirn-Computer-Schnittstellen kombinieren: Sie stimulieren den Nutzer, um ihm eine Rückmeldung zu einer Aktion zu geben, die er mithilfe einer aktiven Gehirn-Computer-Schnittstelle ausgelöst hat [2, S. 809 f.]. So kann eine bidirektionale Schnittstelle bei einer Person, die eine Handprothese nutzt, taktile Empfindungen ersetzen und dadurch den Tastsinn simulieren [1, S. 221 ff.]. Der Nutzer kann fühlen, dass er etwas in der Hand hält und das Gewicht des Gegenstandes einschätzen [41].^25^ Das versetzt ihn in die Lage, seine Prothese intuitiv und mit präziser Feinmotorik zu steuern.

#### Nichtinvasive Methoden versus Neuroimplantate – verbesserte Reha oder Mensch 2.0?

Um eine Gehirn-Computer-Schnittstelle zu nutzen, ist es nicht zwingend notwendig, operativ in das menschliche Gehirn einzugreifen. Auch nichtinvasive Methoden, wie z. B. die Elektroenzephalographie (EEG), können Gehirnsignale über die Schädeldecke messen [1, S. 177 ff.; 2, S. 804].^26^ Eine erwünschte Stimulierung lässt sich ebenfalls von außen durch den Schädel hindurch ins Werk setzen, bspw. mittels transkranieller Magnetstimulierung^27^ oder Gleichstromstimulation.^28^

In Zukunft werden aber voraussichtlich Neuroimplantate – als invasivste Form der Schnittstelle – das Bild prägen. Sie bestehen aus zwei Komponenten: *Mikroelektroden*, die in das Gehirn eingeführt werden, und einem *Neurochip* [1, S. 35 f.], der am Schädel angebracht ist. Dessen Aufgabe ist es, die neurologischen Signale aufzunehmen und (vor) zu verarbeiten; bei *Deep Brain Stimulation* generiert er das Muster der elektrischen Impulse, die dann die Mikroelektroden abgeben [45, S. 5]. Er kann auch mit anderen Geräten kommunizieren – etwa mit einer Armprothese, die Gehirnaktivitätsmuster in die Außenwelt transformiert, oder einem Computer, der die neurologischen Daten analysiert und speichert.^29^

Den Reigen der technischen Möglichkeiten, um organische Denk- mit elektronischen Rechenprozessen zu verbinden, erweitern zahlreiche hochexperimentelle Innovationen aus den Forschungslaboren. So tüfteln Wissenschaftler z. B. an Mikrosensoren, die via Ultraschall neurologische Daten übertragen (sog. *Neural Dust* [2, S. 790; 47]) oder „*Brain-to-Brain-Interfaces*“, mit deren Hilfe sich mehrere Nutzer verbinden und so miteinander kooperieren können.^30^

### IT-Sicherheit von Gehirn-Computer-Schnittstellen

*Hippokrates* hat einmal pointiert: „Die Menschen sollten wissen, dass aus nichts anderem als dem Gehirn Freuden, Wonnen, Gelächter, Spott sowie Kummer, Leid, Verzweiflung und Wehklagen hervorkommen“, (zitiert nach [32, S. 4]). Zu Beginn des 21. Jahrhunderts liest sich die Aussage wie eine prophetische Warnung an potenzielle Nutzer einer Gehirn-Computer-Schnittstelle. Denn so segensreich das Leistungspotenzial der Technologie auch anmutet: Sie ist dem Risiko eines Angriffs von außen in gleicher Weise ausgesetzt wie jedes andere informationstechnische System. Es ist nur eine Frage der Zeit, bis sich Gehirn-Computer-Schnittstellen als sensibles Angriffsziel entpuppen.

Die Dystopie, andere Menschen durch neuronale Manipulation graduell fernzusteuern – bis hin zum Selbstmord wie im Computerspiel *Cyberpunk 2077* – liegt zwar noch in ferner Zukunft. Doch bereits heute bestehen zahlreiche Angriffsvektoren mit zum Teil erheblichem Schadenspotenzial. Bei jeder Gehirn-Computer-Schnittstelle muss IT-Sicherheit^31^ daher von Anfang an mitgedacht werden, um die Vertraulichkeit, Integrität und Verfügbarkeit neurotechnologischer Produkte (*Neurosecurity* [52, S. 2]) zu gewährleisten.^32^

#### Sicherheitslücken

##### *a) Allgemeine Gefahren der IT-Sicherheit von Medizinprodukten*

Schon bisher müssen Hersteller ihre Medizinprodukte zwar in der Regel eingehend prüfen, bevor sie in den Verkehr gelangen.^33^ Dennoch befinden sich in nahezu jedem System Schwachstellen, die sich kompromittieren und für Cyberangriffe ausnutzen lassen [54–57]. Erst kürzlich hat das *Bundesamt für Sicherheit in der Informationstechnologie* (BSI) mehrere vernetzte Medizinprodukte überprüft: Bei allen Produkten entdeckte es – zum Teil gravierende – Sicherheitslücken [58].^34^ Hacker haben wiederholt unter Beweis gestellt, dass sie sich die Kontrolle über sensibelste Medizinprodukte, wie Herzimplantate, verschaffen können [59, 60]. Ist z. B. ein Fernzugriff vorgesehen, öffnet sich unweigerlich ein Tor für einen unbemerkten und unbefugten Zugriff [61]. Aus diesem Grund hat bspw. der ehemalige US-Vizepräsident *Dick Cheney* seinen implantierten Kardioverter-Defibrillator modifizieren lassen, um einem Cyberangriff durch Terroristen vorzubeugen.^35^

Angriffspunkte bietet aber nicht nur das Gerät selbst, sondern auch die digitale Infrastruktur, in die es eingebettet ist. Denn zwischen den IT-Systemen medizinischer Einrichtungen und den dort verwendeten Medizinprodukten bestehen Wechselwirkungen: Eine unsichere Gehirn-Computer-Schnittstelle kann ein Einfallstor in die gesamte IT eines Krankenhauses öffnen [63].^36^

##### *b) Neurosecurity*

Während sich beschädigte klassische Computersysteme typischerweise durch neue austauschen lassen, können Schäden an Gehirn-Computer-Schnittstellen irreversible Folgen zeitigen: Der Angreifer kann nicht nur ein technisches Gerät, sondern mittelbar auch die körperliche sowie mentale Integrität und Gesundheit des Nutzers schädigen.^37^ Die anfallenden neurologischen Daten teilen zudem wichtige Gemeinsamkeiten mit genetischen Daten: Ihnen wohnt ein hoher prognostischer Gehalt für menschliches Verhalten inne; sie legen intime Details frei, welche die betroffene Person häufig nicht einmal selbst kontrollieren kann.^38^ Unbefugte Dritte könnten so Einblicke in emotionale Zustände und ggf. in das neurologische Krankheitsbild eines Nutzers erhalten, die auf anderem Wege nicht beobachtbar sind.^39^

#### Angriffsszenarien

Wege, um Angriffe auf die Vertraulichkeit, Verfügbarkeit, Belastbarkeit und Integrität^40^ von Gehirn-Computer-Schnittstellen zu verüben, gibt es genügend. So ist es denkbar, dass der Angreifer bereits den Stimulus beeinflusst, der beim Opfer bestimmte *neuronale Signalmuster* hervorruft [14, S. 36; 45, S. 8 f.; 73]. Während der *Aufnahme* kann er neuronale Signale stören^41^ oder die aufgenommenen Daten verfälschen.^42^ Ein Eindringling kann ebenfalls die *Verarbeitung* der Rohdaten beim Messen der Gehirnaktivitäten oder ihre *Klassifizierung* manipulieren.^43^ Kontrolliert ein Angreifer etwa den Output der Klassifizierung, übernimmt er das mit der Gehirn-Computer-Schnittstelle gesteuerte Gerät: Ein Patient könnte z. B. die Gewalt über die Bewegungsrichtung und Geschwindigkeit seines Rollstuhls verlieren, den er via Gehirn-Computer-Schnittstelle bedient.^44^ Auch mithilfe einer Schadsoftware lassen sich Geräte, die ein Patient mit einer aktiven Gehirn-Computer-Schnittstellen steuert, übernehmen.^45^

##### *a) Angriffsziele und -folgen*

Die konkreten Spielarten hypothetischer Angriffs- und Schadensszenarien hängen entscheidend vom technischen Design der jeweiligen Gehirn-Computer-Schnittstelle ab – z. B. davon, ob sie „nur“ Gehirnaktivitäten messen und aufnehmen, auch stimulieren, oder ob sie ein Gerät, z. B. eine Prothese, bedienen kann [52, S. 3].

Aktive Gehirn-Computer-Schnittstellen und die mit ihnen vernetzten Prothesen erweisen sich als besonders vulnerables Angriffsziel.^46^ Übernimmt ein Angreifer z. B. die Kontrolle über eine Armprothese, kann er dem Opfer sowie Dritten erhebliche Schäden zufügen. Im Falle einer stimulierenden Gehirn-Computer-Schnittstelle kann ein Angreifer sogar lebensbedrohliche Folgen auslösen,^47^ wenn Neurostimulatoren dem Nutzer elektrische Impulse versetzen.

Generell gilt: Je mehr Funktionen eine Gehirn-Computer-Schnittstelle in sich vereint, desto reichhaltiger ist das Portfolio der denkbaren Angriffsszenarien. Während unbefugte Dritte bei* aktiven Angriffen* in die Funktion der Gehirn-Computer-Schnittstelle eingreifen [14, S. 33; 76, S. 644 ff.], tasten *passive Angriffe* die *Vertraulichkeit*^48^ an, um private Informationen der Nutzer zu erspähen [76, S. 654 ff.]. Dabei können Angreifer zielgerichtet bestimmte Personen anvisieren oder eine bekannte Sicherheitslücke ausnutzen, um betroffene Gehirn-Computer-Schnittstellen „blind“ zu attackieren (sog. *targeted* und* blind* bzw. *mass* oder *opportunistic attacks*) [78, S. 221].

Sollten Schnittstellen es dem Nutzer in Zukunft etwa ermöglichen, nicht nur einen externen Computer zu bedienen, sondern darüber hinaus gleichsam telepathisch mit anderen Personen zu kommunizieren^49^ oder kognitive Fähigkeiten, wie das Erinnerungsvermögen, zu verbessern, eröffnen sich zahlreiche weitere Ansatzpunkte für Beeinträchtigungen.^50^

##### aa) Angriffe auf die Vertraulichkeit

Ein zentraler Angriffspunkt einer Gehirn-Computer-Schnittstelle ist der Neurochip, der die Messdaten verarbeitet sowie mit anderen Computern interagiert und kommuniziert.^51^ Er bündelt in der Regel sensible Daten, die Informationen über den Gesundheitszustand des Patienten an das Tageslicht spülen oder Rückschlüsse auf die Identität des Nutzers zulassen [39, S. 263 f.].^52^

Der Angreifer kann den Nutzer einer aktiven Schnittstelle auch Stimuli aussetzen, um aus den resultierenden Gehirnaktivitäten Schlüsse auf private Informationen zu ziehen.^53^ So haben Forscher in einem Experiment mithilfe frei verkäuflicher EEG-Headsets vierstellige Geheimzahlen (PINs) in Erfahrung gebracht [81, S. 147].^54^ Künftig können Angreifer auf diese Weise womöglich Informationen über die politische oder religiöse Ausrichtung, Erinnerungen oder emotionale Reaktionen extrahieren [82, S. 387 ff.; 83, S. 3, 22 f.] sowie Lügen detektieren [84, S. 366 ff.] – es entstünde eine „*Brain Spyware*“ [14, S. 33; 85, S. 419 ff.; 86]. Das macht Daten, die Gehirn-Computer-Schnittstellen zutage fördern, für viele Anwendungen attraktiv – bis hin zu Maßnahmen der Terrorismusbekämpfung oder Strafermittlung [84, S. 351 ff.; 87].^55^

Die Informationen, die ein Angreifer erlangt, lassen sich auf vielfältige Weise missbrauchen, insbesondere monetarisieren. Gesundheitsbezogene Informationen kann der Angreifer bspw. verwenden, um den Nutzer bzw. Patienten zu erpressen [76, S. 660; 89] oder um sie im Darknet feilzubieten.^56^ Je nach Art der Informationen können sie für einen Identitätsdiebstahl oder Krankenversicherungsbetrug bzw. -missbrauch [90] zum Einsatz kommen.^57^ Zudem lassen sich z. B. umfangreiche Aktivitätenprofile der betroffenen Person erstellen [92, S. 3].

Nicht zuletzt könnte der Angreifer in „Lauschangriffen“ die Kommunikation zwischen verschiedenen Komponenten der Gehirn-Computer-Schnittstelle „abhören“ und aufzeichnen [39, S. 263]. Denkbar ist es bspw., Menschen unbefugt zu scannen, um festzustellen, ob sie Implantate in sich tragen und welches Modell sie nutzen [4, S. 193; 39, S. 263].^58^ Dadurch lassen sich Rückschlüsse auf den Gesundheitszustand einer Person ziehen oder passgenaue Angriffsszenarien entwickeln. Hat ein Angreifer etwa die Modell- oder Seriennummer des Geräts erspäht und bereits bekannte Sicherheitslücken ausfindig gemacht, kann er weitere (auch aktive) Angriffe auf die Gehirn-Computer-Schnittstelle leichter durchführen.^59^

##### bb) Angriffe auf die Verfügbarkeit und Belastbarkeit

Angriffe auf die *Verfügbarkeit*^60^ eines Systems beeinträchtigen die Funktionsfähigkeit einer Gehirn-Computer-Schnittstelle [52, S. 2; 72, S. 6; 77, Rn. 38].^61^ Die Neuroprothese eines beinamputierten Menschen funktioniert dann beim Gehen nicht mehr, der Nutzer stürzt und verletzt sich [52, S. 3]; eine stimulierende Schnittstelle kann einen Epilepsieanfall nicht mehr verhindern oder abmildern [52, S. 3; 78, S. 221.]. Ein Angreifer kann bspw. die Gehirn-Computer-Schnittstelle mit massenhaften Anfragen überlasten (sog. *Denial-of-Service*[DoS]-Angriffe), um sie gezielt unter der Last der Anfragen kollabieren zu lassen [51, S. 41; 72, S. 113].^62^ Dann kann sie legitime Anfragen und Prozesse nicht mehr durchführen. DoS-Angriffe können ebenfalls die *Belastbarkeit* einer Gehirn-Computer-Schnittstelle strapazieren: Das Gerät ist dann nicht hinreichend robust, um Gefahrenlagen zu bewältigen; insbesondere kann es die notwendige Leistungsfähigkeit nicht länger aufrechterhalten oder zügig wiederherstellen.^63^

##### cc) Angriffe auf die Integrität

Die wohl schwerwiegendsten Attacken auf Gehirn-Computer-Schnittstellen sind solche, die auf ihre Integrität zielen: Ein Angreifer manipuliert in diesem Fall die Informationen bzw. die Kommunikation mit anderen Geräten oder beeinträchtigt die Funktionsfähigkeit des Systems insgesamt [14, S. 33; 72, S. 5 f.; 76, S. 644 ff.].^64^ So lassen sich Geräteeinstellungen, Befehle oder Daten verändern [39, S. 264; 52, S. 2]^65^ sowie von Angreifern manipulierte Updates injizieren [45, S. 10; 98, S. 132 f.], um die Steuerung einer Prothese zu übernehmen (*Hijacking* [74, S. 123 f.] bzw. *Brainjacking* [78]) oder ein fehlerhaftes Feedback an die Schnittstelle zu übermitteln [39, S. 265; 52, S. 3]. Besonders brenzlig wird es, wenn Angreifer in neurologische Abläufe eingreifen und dadurch Schmerzen, Emotionen oder einen Verlust der Impulskontrolle herbeiführen, die empfindliche Schäden nach sich ziehen [39, S. 266; 52, S. 3; 78, S. 221; 92, S. 3].^66^ In ferner Zukunft könnte es gar möglich sein, dass ein Angreifer die Gedanken eines anderen Menschen beeinflusst und ihn so zu bestimmten Handlungen verleitet.

##### *b) Angriffsvektoren und häufige Schwachstellen*

Besonders groß sind Angriffsflächen für Cyberattacken,^67^ wenn Geräte drahtlos^68^ vernetzt sind [24, S. 829, 833 ff.; 100, S. 14 ff.; 101, S. 116 ff.]. Viele medizinische Implantate sind zwingend auf eine drahtlose Verbindung angewiesen, weil sie im Körper eingepflanzt und nicht effektiv auf anderen Wegen ansteuerbar sind.

Um die Informationen, die ein Implantat erhebt, speichern und analysieren zu können (z. B. um das Implantat auf seine Funktionsfähigkeit hin zu überprüfen oder den Therapieerfolg zu überwachen), interagiert die Schnittstelle mit einem PC oder Smartphone. Veraltete Software, insbesondere nicht mehr aktuelle Betriebssysteme,^69^ können dann als Einfallstor dienen, um Geräte zu attackieren [104, S. 404 ff.; 105]. Zahlreiche Medizinprodukte senden Daten an einen Home-Monitor, der diese sammelt und drahtlos in ein Repositorium hochlädt, sodass der behandelnde Arzt diese auf einer Webseite einsehen kann [39, S. 256].^70^ Bei *Deep Brain Stimulation*^71^ kommt das Gerät, das die Abgabe des elektrischen Pulses zur Stimulierung regelt, im Brustbereich zum Einsatz und kommuniziert mit den Mikroelektroden im Gehirn, die neuronale Aktivitäten messen [39, S. 256]. Übertragen Medizinprodukte Daten unverschlüsselt, wie etwa einige implantierte Kardioverter-Defibrillatoren^72^, können Unbefugte sie mit einer Funkausrüstung mitlesen [92, S. 5].^73^

Wenn Gehirn-Computer-Schnittstellen perspektivisch Daten drahtlos mit einem Smartphone über eine App austauschen, potenziert sich das Risiko [92, S. 5].^74^ Denn nicht nur von der Schnittstelle selbst gehen dann Gefahren aus, sondern auch von dem mit ihr *verbundenen Gerät*, z. B. einem Smartphone [24, S. 831].^75^

## Rechtsrahmen für Gehirn-Computer-Schnittstellen

Die berechtigte Sorge vor Cyberangriffen auf Medizinprodukte [108, 109]^76^ ruft allerorten Regierungen und Behörden auf den Plan, effektive und handhabbare Regelungen und Anforderungen für die Cybersicherheit von Medizinprodukten zu entwickeln.^77^

Auf den ersten Blick drängt sich das Strafrecht als Abschreckungswaffe par excellence auf, um Cyberangriffen entgegenzuwirken.^78^ In praxi entpuppt es sich jedoch als vergleichsweise stumpfes Schwert. Da Angriffe für die Cybersicherheit örtlich entkoppelt erfolgen können, entziehen sich (international operierende oder sich gekonnt verschleiernde) Akteure bislang typischerweise erfolgreich dem Zugriff der Strafverfolgungsbehörden [111, S. 1129 ff.]:^79^ Sie agieren meist von Orten aus, in denen sie keine Strafverfolgung fürchten müssen, oder unter der Obhut eines Staates.^80^ Bei Gefahren für hochrangige Rechtsgüter wie Leben, Körper, Gesundheit und mentale Integrität, genügt die repressive Konzeption des Strafrechts ohnedies nicht als Schutzinstrument. Es sind zwingend auch präventive Ansätze und Strategien geboten: Wirksame Schutzmaßnahmen müssen Angreifern den Zugriff von vornherein unmöglich machen oder zumindest wesentlich erschweren.

### Verfassungsrechtliche Schutzgüter

Ein Regelungskonzept gegen Cyberangriffe muss sich bruchfrei in den verfassungsrechtlichen Rahmen einbetten, den das Grundgesetz zieht. Dieses schützt den Einzelnen gegen den Zugriff Dritter auf seine Gehirn-Computer-Schnittstellen in unterschiedlichen grundrechtlichen Tatbeständen.

#### Betroffene Grundrechte

##### *a) Schutz der Privatsphäre: personenbezogene Daten und IT-Systeme (Art. 7 und Art. 8 GRCh, Art. 2 Abs. 1 i.* *V.* *m. Art. 1 Abs. 1 GG); Telekommunikationsgeheimnis (Art. 10 Abs. 1 GG)*

Erbeuten Cyberangriffe Informationen zum (neurologischen oder psychischen) Gesundheitszustand oder zu emotionalen Reaktionen, legt das intimste Bereiche der Privatheit des Nutzers offen^81^ und tangiert dadurch die *informationelle Selbstbestimmung*. Diese verbürgt jedem Grundrechtsträger das Recht, grundsätzlich selbst zu bestimmen, ob und innerhalb welcher Grenzen er persönliche Lebenssachverhalte offenbart (Art. 2 Abs. 1 GG i. V. m. Art. 1 Abs. 1 GG bzw. Art. 7 und 8 GRCh und Art. 8 EMRK).^82^ Die grundrechtliche Wertung wirkt auch in privatrechtliche Beziehungen hinein. Ihr Schutzgehalt versagt es Dritten, die Persönlichkeit der betroffenen Person zu registrieren, zu katalogisieren und ein umfassendes Persönlichkeitsprofil zu erstellen.^83^

Das *Grundrecht auf Gewährleistung der Vertraulichkeit und Integrität informationstechnischer Systeme* (sog. IT-Grundrecht) gewährt einer anderen Komponente des Persönlichkeitsrechts besonderen grundrechtlichen Schutz: Es richtet seinen Schutzradius nicht nur auf einzelne Daten aus, sondern bewahrt das gesamte System davor, dass Unbefugte eindringen und es modifizieren; es schützt sowohl die Unversehrtheit der Daten als auch die Funktionsweise des Systems.^84^ Soweit ein IT-System Kommunikationsinhalte an *Dritte* überträgt, tritt ergänzend das *Telekommunikationsgeheimnis*^85^ als Schutzrecht hinzu: Es verbürgt die Vertraulichkeit der Inhalte und Umstände individueller Kommunikationsvorgänge,^86^ solange sie aufgrund des Übertragungsvorgangs erhöhten Zugriffsgefahren ausgesetzt sind.^87^

##### *b) Angriffe auf die körperliche und „mentale“ Integrität*

Fügt ein Cyberangriff den Nutzern einer Schnittstelle Schmerzen zu oder verursacht er Schäden im Gehirn, greift dies in die körperliche Integrität und ggf. das Recht auf Leben (Art. 2 Abs. 2 S. 1 GG) ein [24, S. 836 f.; 119, Rn. 116; 125, Rn. 55; 126, S. 467]^88^ – ebenso, wenn ein Angreifer auf ein Neuroimplantat einwirkt, auf das ein Patient angewiesen ist [125, Rn. 56; 128, S. 236 ff.].^89^

Manipulationen, welche die *„mentale Integrität“*^90^ betreffen, lassen sich schwerer in das grundrechtliche Raster einordnen. Sie beeinflussen Gehirnaktivitäten und rufen emotionale Zustände oder sonstige Reaktionen hervor [83, S. 21 ff.]. Auch das Verhalten, die Persönlichkeit oder Identität können sie verändern [74, S. 125 ff.; 83, S. 24 ff.; 131]. Betrachtet man solche Steuerungen der Gehirnaktivitäten rein neurowissenschaftlich, handelt es sich um einen Eingriff in körperliche Vorgänge (Art. 2 Abs. 2 S. 1 GG).^91^ Berührt eine Gehirnmanipulation die Grundlage menschlicher Selbstwahrnehmung sowie die Konstituierung des Ichs, erschöpft sie sich nicht in einem körperlichen Eingriff.^92^ Sie kann einerseits die Menschenwürde^93^ und andererseits das allgemeine Persönlichkeitsrecht (Art. 2 Abs. 1 GG i. V. m. Art. 1 Abs. 1 GG) berühren.^94^

##### *c) Autonomie und Handlungsfreiheit*

Wer die Kontrolle über seine Gehirn-Computer-Schnittstelle und ggf. über eine hierdurch gesteuerte Prothese verliert, büßt mehr ein als nur seine Daten und körperliche Integrität. Im Extremfall geht er seiner Fähigkeit verlustig, Entscheidungen im Einklang mit seinen Wünschen und Absichten zu treffen und diese selbstbestimmt in die Tat umzusetzen [78, S. 223; 134, S. 72].

Die Kategorie „Autonomie“^95^ ist der deutschen Grundrechtsdogmatik zwar nicht als solche vertraut. Die allgemeine Handlungsfreiheit (Art. 2 Abs. 1 GG) und das allgemeine Persönlichkeitsrecht (Art. 2 Abs. 1 GG i. V. m. Art. 1 Abs. 1 GG), das den Geltungsanspruch des Individuums in der sozialen Welt schützt,^96^ decken allerdings zentrale Aspekte der menschlichen Autonomie ab. Dies gilt insbesondere für solche Konstellationen, in denen die Gehirn-Computer-Schnittstelle medizinisch notwendig bzw. rehabilitativ ist, der Patient also nur mit ihrer Hilfe überhaupt in der Lage ist, sein Leben selbstbestimmt zu führen.^97^ Bei Schnittstellen, die motorische Fähigkeiten wiederherstellen, läuft der Patient bspw. Gefahr, seine Bewegungsfreiheit einzubüßen.^98^

##### *d) Eigentum*

Schaltet ein Angreifer eine Gehirn-Computer-Schnittstelle aus oder zerstört ihre Funktionsfähigkeit,^99^ beeinträchtigt das ihren Nutzer in seinem Eigentumsrecht (Art. 14 Abs. 1 GG).^100^ Das Gleiche gilt, wenn Cyberangriffe die Gehirn-Computer-Schnittstelle mit Anfragen überlasten, bis sie ihre Funktionen nicht mehr erfüllen kann.^101^ Auch in diesen Fällen entzieht der Angreifer dem Eigentümer die Nutzungsmöglichkeit.

#### Reichweite der Schutzpflicht

Das vielschichtige Gewährleistungspotpourri der Grundrechte vermittelt dem Einzelnen nicht nur ein *Abwehrrecht* gegen staatliche Zugriffe, sondern konstituiert auch eine staatliche* Schutzpflicht* gegen das Wirken Privater [120, S. 96 ff.; 142, S. 3535]: Kraft der objektivrechtlichen Dimension der Grundrechte ist der Staat verpflichtet, sich „schützend und fördernd“ vor sie zu stellen.^102^ Das gilt für die Integrität und Vertraulichkeit von IT-Systemen in besonderer Weise: Der Staat kann diese nur dann angemessen gewährleisten, wenn er dem Schutzgehalt auch gegenüber Privaten zur Wirksamkeit verhilft [120, S. 96 ff.; 142, S. 3535; 144, S. 114 ff.].^103^

Seinem Ausgestaltungsauftrag ist der Gesetzgeber im Ansatz bspw. durch die Strafvorschriften für unbefugtes Ausspähen (§ 202a StGB) und Abfangen von Daten (§ 202b StGB)^104^ sowie durch die Tatbestände der Datenveränderung (§ 303a StGB) und Computersabotage (§ 303b StGB)^105^ nachgekommen.^106^ Mit Blick auf die elementaren Risiken, die von Angriffen auf Gehirn-Computer-Schnittstellen ausgehen, ist die Rechtsordnung jedoch auch aufgerufen, hohe Anforderungen an die Cybersicherheit^107^ zu stellen, um dystopische „Brainhacks“ möglichst zu verhindern, oder wenigstens zu erschweren bzw. schnellstmöglich zu unterbinden. Das Datenschutz- und IT-Sicherheitsrecht bis hin zu den Vorgaben im Medizinprodukterecht sind Ausdruck dieses Schutzauftrags.^108^

Die staatliche Schutzpflicht ist aber nicht grenzenlos. Sie stößt an ihre Schranken, wenn sich Nutzer einer Gehirn-Computer-Schnittstelle eigenverantwortlich selbst gefährden, um die eigene Leistungsfähigkeit zu optimieren (sog. *Neuroenhancement*). Die Freiheitsrechte (und damit die Schutzpflicht des Staates) sind nicht als ein aufgedrängtes Schutzgut konzipiert, das den Einzelnen vor sich selbst schützt:^109^ Der Staat darf sie dem Einzelnen nicht ohne Weiteres aufnötigen, soweit die Selbstgefährdung nicht zugleich die Allgemeinheit intensiv beeinträchtigt [125, Rn. 84; 126, S. 469], z. B. weil sie dem Gemeinwesen hohe Gesundheitskosten aufbürdet.^110^ Dem Einzelnen bleibt es deshalb im Grundsatz unbenommen, sich selbst durch Neuroenhancement zu gefährden oder zu schädigen sowie in Einwirkungen einzuwilligen [126, S. 467].^111^ Das Recht, Neuroenhancement zu betreiben, ist Teil des Schutzgehalts der allgemeinen Handlungsfreiheit.^112^

Umgekehrt darf das Gemeinwesen seine Hilfe dem Einzelnen nicht ohne Weiteres deshalb vorenthalten, weil er sich zum Zwecke der Selbstoptimierung selbst gefährdet hat. Wer sich selbst in Gefahr gebracht hat, den darf der Staat nicht gleichsam fallen lassen und ihn seinem Schicksal überlassen. So greift die staatliche Schutzpflicht auch für denjenigen, der Drogen konsumiert^113^ oder sich einer nicht medizinisch indizierten Schönheitsoperation unterzogen hat.^114^ Die Schutzpflicht endet erst, wenn der sich selbst Gefährdende wider besseres Wissen handelt und auf die Hilfe anderer spekuliert [125, Rn. 85].^115^

Als Ausdruck seiner Schutzpflicht ist der Staat im Ergebnis gehalten, bei nichtmedizinischen Neurotechnologieprodukten,^116^ welche die Gesundheit beeinträchtigen können, durch Regulierung für ein hinreichendes Maß an IT-Sicherheit zu sorgen. Wenn der Einzelne die Risiken seiner Selbstgefährdung durch sog. „Do-it-yourself“-Produkte, die Privatpersonen zusammenbasteln und anwenden [10],^117^ zu spät erkennt und sich von den Folgen befreien möchte, hat der Staat ihm ein Hilfsangebot zu machen.^118^

### Einfachrechtliche Anforderungen

Ein spezifisches einfachgesetzliches Rechtsregime für Gehirn-Computer-Schnittstellen hat der Gesetzgeber bislang nicht entfaltet. Das Datenschutzrecht (1.), das Medizinprodukterecht (2.) als auch allgemeine Vorschriften des IT-Sicherheitsrechts (3.) stecken jedoch einen groben normativen Rahmen ab.

#### Das Datenschutzrecht als allgemeiner IT-sicherheitsrechtlicher Überbau

##### *a) Verarbeitung personenbezogener Daten*

Gehirnsignale, die Gehirn-Computer-Schnittstellen aufnehmen, machen eine Person identifizierbar. Als personenbezogene Daten (Art. 4 Nr. 1 DSGVO) unterliegen sie dem Regelungsanspruch der DSGVO [157, S. 388 f.; 158, S. 107 ff.; 159, S. 5]. Sie gehören zudem einer besonderen Sensibilitätskategorie des Datenschutzrechts an. Denn sie geben typischerweise Auskunft über die körperliche und geistige Gesundheit einer natürlichen Person und sind damit Gesundheitsdaten (Art. 4 Nr. 15 DSGVO), die den besonders hohen Rechtfertigungshürden des Art. 9 Abs. 1 DSGVO unterliegen.^119^ So werten manche Apps oder Spiele, die mit Gehirn-Computer-Schnittstellen interagieren, bspw. Informationen zum Stresspegel aus^120^ und schließen hieraus auf die (psychische) Gesundheit des Nutzers. Selbst bei Geräten, die nicht im medizinischen Bereich zum Einsatz kommen, können neurologische Daten ggf. Informationen über den gegenwärtigen oder künftigen körperlichen oder psychischen Gesundheitszustand des Nutzers preisgeben.^121^ Darüber hinaus lassen sich Gehirnaktivitätsmuster als physiologische Merkmale einsetzen, um Personen automatisiert zu identifizieren und zu authentifizieren.^122^ Auch die Verarbeitung solcher biometrischer Daten (Art. 4 Nr. 14 DSGVO) muss sich an Art. 9 Abs. 1 DSGVO messen lassen.

##### *b) Datensicherheit*

Der Regelungsanspruch der DSGVO erschöpft sich keineswegs in *datenschutz*rechtlichen Geboten. Sie formuliert vielmehr auch Anforderungen an die *Datensicherheit*: Der Verantwortliche muss (ebenso wie der etwaige Auftragsverarbeiter) geeignete technische und organisatorische Maßnahmen treffen, um ein dem Risiko angemessenes Sicherheitsschutzniveau zu gewährleisten (Art. 32 Abs. 1 DSGVO).^123^ Er hat insbesondere die Vertraulichkeit, Integrität, Verfügbarkeit und Belastbarkeit der datenverarbeitenden Systeme sicherzustellen (Art. 32 Abs. 1 Hs. 2 lit. b). Nach einem Zwischenfall muss der Verantwortliche ferner in der Lage sein, die Verfügbarkeit der Daten rasch wiederherzustellen (lit. c). Er muss (ähnlich der Datenschutzfolgenabschätzung nach Art. 35 DSGVO) ein Konzept zum Datensicherheitsmanagement erarbeiten [77, Rn. 59 ff.; 167, Rn. 23].^124^ Dieses Schutzkonzept hat er im Anschluss regelmäßig zu überprüfen (lit. d). Das einzuhaltende Schutzniveau muss sich an dem Risiko ausrichten, das sich mit der Verarbeitung verbindet.^125^

Da Gehirn-Computer-Schnittstellen in der Regel hochsensible biometrische und Gesundheitsdaten verarbeiten, geht von ihnen selbst dann ein hohes Risiko aus, wenn die Wahrscheinlichkeit eines Angriffs gering ist.^126^ Sie müssen daher höchsten Ansprüchen genügen, um Zugriffe möglichst zu vermeiden. Die zu ergreifenden Maßnahmen müssen dem Stand der Technik entsprechen, d. h. auf gesicherten Erkenntnissen der Wissenschaft und Technik beruhen, die sich in der Praxis bewährt haben [77, Rn. 56a; 96, Rn. 18]. Seine Anforderungen entwickeln sich im Gleichschritt mit technologischen Innovationen dynamisch fort [77, Rn. 56b, 57]. Einen ersten Konkretisierungsversuch wagen etwa die ISO 27000-Normenreihe^127^ sowie das IT-Grundschutz-Kompendium^128^ des BSI [77, Rn. 57; 170].

##### aa) Verschlüsselung

Eine Basismaßnahme, um sicherzustellen, dass eine Gehirn-Computer-Schnittstelle mit anderen Geräten vertraulich kommuniziert, ist deren Verschlüsselung [24, S. 829; 76, S. 653; 100, S. 16]: Daten sind während der Übertragung („in motion“) und im ruhenden Zustand („at rest“) mit einer geeigneten Verschlüsselungsmethode zu schützen (Art. 32 Abs. 1 lit. a DSGVO) [77, Rn. 34 ff.; 171, S. 74 ff.].^129^

##### bb) Authentifikationserfordernis und Zugriffsverwaltung

Um einen unbefugten Fremdzugriff zu verhindern, sollte das System den Zugriff auf eine Gehirn-Computer-Schnittstelle nur nach vorheriger Authentifikation ermöglichen [77, Rn. 35d].^130^ Eine Benutzerzugriffsverwaltung sollte den Berechtigten (restriktiv) Privilegien und Befugnisse einräumen [92, S. 7].

Während bei EEG-Headsets häufig eine individuelle Nutzername-Passwort-Kombination^131^ ausreicht, ist bei Neuroimplantaten und Neurostimulatoren eine Zwei-Faktor-Authentifizierung^132^ geboten. Wer seinen Rollstuhl per Gehirn-Computer-Schnittstelle lenkt, sollte sich nicht einfach via Bluetooth „koppeln“ können, sondern diesen z. B. durch seine Gehirnwellen in Verbindung mit einer Smartphone-App ansteuern können.

##### cc) Angriffserkennung

Um die Gefahren zu minimieren, die von dem Gerät ausgehen, das mit der Schnittstelle verbunden ist, sollten Anbieter die Gehirn-Computer-Schnittstelle mit einer Detektions-Software ausstatten [76, S. 652; 77, Rn. 36a].^133^ Auf diese Weise lassen sich untypische Aktivitäten oder Schadsoftware erkennen.^134^ Hilfreich ist es zudem, alle Aktivitäten und Zugriffe auf die Gehirn-Computer-Schnittstelle in einer Logdatei festzuhalten und diese regelmäßig auf Unregelmäßigkeiten zu überprüfen [92, S. 7]. Insbesondere sicherheitsrelevante Ereignisse sind zu protokollieren [77, Rn. 39].^135^ DoS-Angriffe kann die Gehirn-Computer-Schnittstelle bspw. bewältigen, indem sie als Gegenmaßnahme Anfragen filtert^136^ bzw. zurückstellt und ihre Kernfunktionen (das Aufnehmen und Auswerten von Gehirnsignalen) priorisiert.

##### dd) Security by Design

Die Gehirn-Computer-Schnittstelle sollte sich bei ihren Datenzugriffen entsprechend dem Gebot der Datenminimierung (Art. 5 Abs. 1 lit. c DSGVO) auf die notwendigen Informationen und Verarbeitungsschritte beschränken [100, S. 16; 174, S. 89 ff.; 175; 176, Rn. 34 ff.]. Das verkleinert die Angriffsfläche. Bspw. kann die Gehirn-Computer-Schnittstelle neuronale Signale, die für die spezifische Aufgabe erforderlich sind, selbst vorverarbeiten, bevor sie bspw. auf IT-Systeme Dritter übertragen werden (sog. *Edge Computing*) [177, v. a. S. 642]. Rohdaten oder Daten, die nicht explizit für die jeweilige Aufgabe erforderlich sind, muss sie dann nicht übermitteln oder speichern (und sie dadurch Sicherheitsrisiken exponieren).^137^ Kraft ihres technischen Zuschnitts („by Design“) sollte die Gehirn-Computer-Schnittstelle so weit wie möglich vermeiden, personenbezogene Daten zu übermitteln: IP-Adressen oder die Modell- bzw. Device-ID sollte das Gerät standardmäßig entfernen, bevor es Daten an eine Plattform übermittelt [100, S. 16].

##### ee) Defense in Depth und Vorfallsmanagement

IT-Sicherheit ist kein Produkt, sondern ein Prozess [178]: Softwaresysteme sind dynamisch und verändern sich stetig. Hacker^138^ finden zudem immer neue Schlupfwege in vermeintlich sichere Systeme. Durch technischen Datenschutz allein wird es daher nicht gelingen, Angriffe auf vernetzte Geräte zuverlässig abzuwehren.^139^ Kein IT-System genießt überdies absoluten Schutz [171, S. 2 f.; 174, S. 94; 181]. Eine gute Sicherheitsarchitektur besteht daher aus vielen, stetig weiterzuentwickelnden Komponenten und Schichten, die einen hinreichenden Schutz sicherstellen (*Defense in Depth*).^140^

Einem Angreifer genügt bereits *eine* Schwachstelle. Eine effektive Verteidigungsstrategie muss daher rundum schützen [51, S. 71]. Die Schutzmaßnahmen sollten auch aus diesem Grund nicht nur unmittelbar an der Schnittstelle selbst, sondern ggf. auch an den mit ihr verbundenen Geräten ansetzen.^141^

Eine entscheidende Rolle bei dem Schutzmaßnahmenpaket kommt dem Vorfallsmanagement zu. Insbesondere Sicherheitsupdates^142^ tragen dazu bei, Sicherheitslücken so schnell wie möglich zu erkennen und zu beheben. Ein besonderes Augenmerk sollte dem verwendeten Open-Source-Code und Programmbibliotheken gelten, die Schwachstellen enthalten könnten bzw. unter Umständen einer Aktualisierung bedürfen [71, S. 22 f.; 101, S. 131]. Regelmäßige Penetrationstests^143^ und Red-Team-Einsätze^144^ helfen, die Sicherheitsmaßnahmen zu überprüfen und zu bewerten (vgl. Art. 32 Abs. 1 Hs. 2 lit. d DSGVO).^145^

Ist das Kind der IT-Sicherheit erst einmal gleichsam in den Brunnen gefallen, ist es also zu einem Sicherheitsvorfall gekommen, der Unbefugten Zugriff auf die personenbezogenen Daten eröffnet hat, muss der Verantwortliche dies der Aufsichtsbehörde (Art. 33 Abs. 1 DSGVO, § 65 BDSG) sowie der betroffenen Person^146^ (Art. 34 Abs. 1 DSGVO, § 66 BDSG) melden. Er sollte zudem ein Vorfalls- und Notfall-Management einrichten, das darauf gerichtet ist, die Schwachstelle zu untersuchen und zu beheben [51, S. 153 ff.], um im Ernstfall rasch auf Vorkommnisse reagieren zu können (Art. 32 Abs. 1 Hs. 2 lit. c DSGVO).^147^

##### *c) Adressat der Pflichten*

Am zuverlässigsten kann der *Hersteller* des Produkts ein hohes Sicherheitsniveau der Gehirn-Computer-Schnittstelle verbürgen. Die sicherheitsrechtlichen Pflichten der Art. 32 ff. DSGVO treffen allerdings nicht den Hersteller eines Produkts, sondern alleine den datenschutzrechtlich *Verantwortlichen*, also denjenigen, der tatsächlich oder rechtlich über die Zwecke und Mittel der Datenverarbeitung entscheidet (Art. 4 Nr. 7 DSGVO) [185, Rn. 170].

Verantwortlicher im datenschutzrechtlichen Sinn ist der Hersteller nur dann, wenn er für die Schnittstelle eine App oder eine Cloud vorhält, die Daten auf die Server des Anbieters hochlädt und dort z. B. analysiert, sodass er die relevanten personenbezogenen Daten verarbeitet. Seine Pflichten aus Art. 32 ff. DSGVO erstrecken sich in diesem Fall aber nur auf den *Verarbeitungsvorgang* selbst, nicht auf die *Herstellungseigenschaften* des Produkts, insbesondere die technischen Eigenschaften der Schnittstelle [77, Rn. 27; 186, S. 77].^148^ Diese sind nicht Gegenstand des datenschutzrechtlichen Pflichtenradars. Das Regulierungsportfolio der DSGVO hat an dieser wichtigen Stelle eine Lücke: Denjenigen, der die Weichenstellungen für die Gehirn-Computer-Schnittstelle trifft, adressiert sie grundsätzlich nicht. Effektiv anonymisieren, pseudonymisieren sowie verschlüsseln kann aber nur derjenige, dem dafür überhaupt die technischen Möglichkeiten zur Verfügung stehen. Ein App-Anbieter muss seine Software als Verantwortlicher mithin so gestalten, dass sie Datensicherheit gewährleistet, selbst wenn er keinen unmittelbaren Einfluss auf die eingesetzte Hardware hat. Im Bereich der „Do-it-yourself“*-*Produkte und Analysetools entscheidet dagegen der Nutzer als Verantwortlicher typischerweise selbst, welche Daten seine Gehirn-Computer-Schnittstelle erhebt und verarbeitet.

#### Medizinprodukterecht

Kommen Gehirn-Computer-Schnittstellen im Gesundheitswesen zum Einsatz, zieht die Medizinprodukte-Verordnung (MPVO) ergänzende normative Leitplanken ein, die Sicherheitsanforderungen etablieren.^149^ Seit dem 26. Mai 2021 ist sie in der gesamten EU unmittelbar anwendbar.^150^

##### *a) Gehirn-Computer-Schnittstellen als Medizinprodukte*

Die MPVO erstreckt sich grundsätzlich auf Produkte, die für Menschen bestimmt sind und einen spezifischen medizinischen Zweck erfüllen: Sie sollen Krankheiten oder Behinderungen diagnostizieren, überwachen, behandeln oder lindern (Art. 2 Nr. 1 MPVO).

Sofern eine Gehirn-Computer-Schnittstelle eine medizinische Zweckbestimmung aufweist, fällt sie daher typischerweise in den Anwendungsbereich der Verordnung. Diese Voraussetzungen erfüllen etwa dauerhaft implantierte^151^*Deep-Brain-*Stimulatoren oder andere Neuroimplantate, die Parkinson-Tremores oder Epilepsie behandeln sollen.^152^ Auch EEG-Headsets mit medizinischer Zweckbestimmung sind Medizinprodukte.^153^ Die dazugehörige Software ist Teil des Produkts, da sie dieses steuert und dessen Anwendungen beeinflusst.^154^ Ein *eigenständiges* Medizinprodukt ist Software demgegenüber nur dann, wenn sie Informationen zu Entscheidungen für diagnostische oder therapeutische Zwecke liefert oder physiologische Prozesse kontrolliert.^155^ Dies gilt bspw. für Apps [192, S. 198], welche die Funktionen und Anwendungsmöglichkeiten der Gehirn-Computer-Schnittstelle erweitern. Apps oder Anwendungen, die Daten lediglich speichern, archivieren, kommunizieren oder anzeigen, erfasst die MPVO hingegen nicht [192, S. 198].

##### aa) Medizinische Zweckbestimmung

Ob ein Produkt eine medizinische Zweckbestimmung aufweist, bestimmt sich (anders als man auf den ersten Blick vermuten könnte) nicht danach, welchen Zweck die *Verbraucher* einer Gehirn-Computer-Schnittstelle im Rahmen ihres Konsumverhaltens unterlegen. Entscheidend ist allein die Zweckbestimmung des *Herstellers* („dem Hersteller zufolge“).^156^

Es verwundert deshalb nicht, dass viele Hersteller ihre EEG-Headsets unter ausdrücklichem Ausschluss einer medizinischen Zweckbestimmung verkaufen.^157^ Solche Freizeit- bzw. Verbraucherprodukte, die u. a. dazu dienen, Konzentration oder Stress zu überwachen, sind keine Medizinprodukte i. S. der MPVO.^158^ Diese „Wellness“-Sparte von EEG-Headsets, mit denen Nutzer ihre Gehirnaktivitäten (i. S. des „Quantified Self“^159^) ohne medizinische Indikation selbst überwachen, dient der allgemeinen Gesundheitsförderung, die sich auf Lifestyle-Optimierung und kleinere Befindlichkeitsstörungen bezieht [194, Rn. 4].^160^

##### bb) Produkte ohne medizinische Zweckbestimmung

Auf Gehirn-Computer-Schnittstellen ohne medizinische Zweckbestimmung erstreckt sich die MPVO ausnahmsweise dann, wenn diese das Gehirn (nichtinvasiv) transkraniell (also durch die Schädeldecke hindurch) stimulieren (Art. 1 Abs. 2 MPVO i. V. m. Anhang XVI Nr. 6 MPVO). Damit will der Unionsgesetzgeber den Risiken begegnen, die von einer solchen besonderen Art der Stimulation ausgehen, und Produkte, die manchmal zu medizinischen Zwecken und manchmal für *Enhancement* zur Anwendung kommen, im Ergebnis den gleichen Anforderungen unterwerfen [44, S. 78 ff.].^161^ Bei ihnen legt es die MPVO also bewusst nicht in die Hand der Hersteller, durch eigene Zweckbestimmung darüber zu befinden, ob die strengen Vorschriften des Rechts der Medizinprodukte zur Anwendung kommen.

Mit Blick auf Produkte, die sich derzeit noch im Forschungsstadium befinden, zeigen sich indes die ersten normativen Lücken der MPVO. So möchte *Neuralink* ein multifunktionales Neuroimplantat anbieten, das nicht nur neuronale Aktivitäten aufnehmen, sondern auch Neuronen-Cluster stimulieren kann. Solche (futuristischen) Neuroimplantate kämen weder zwingend zu medizinischen Zwecken zum Einsatz [194, Rn. 5] noch nähmen sie eine transkranielle (sondern eine *intra*kranielle, innerhalb des Gehirns erfolgende) Stimulierung des Gehirns vor.^162^ Der Normgeber hatte scheinbar nur die (nichtmedizinischen) Stimulierungen *durch* die Schädeldecke im Blick, nicht aber die invasivere Stimulierung *innerhalb* des Schädels. Da die Liste in Anhang XVI Ausnahmecharakter hat, lässt sie sich auch nicht durch teleologische Extension um die risikoreichere intrakranielle Stimulierung erweitern.

Anhang XVI Nr. 2 MPVO erstreckt das Regelungsregime der MPVO zwar auch auf solche Produkte, „die dazu bestimmt sind, durch chirurgisch-invasive Verfahren zum Zwecke der Modifizierung der Anatomie […] in den menschlichen Körper eingeführt zu werden“.^163^ Implantierte Mikroelektroden, wie sie z. B. *Neuralink* anvisiert, verändern die Gehirnanatomie oder -struktur jedoch nicht – abgesehen von neuroplastischen Veränderungen bei der Implantation und „Bedienung“ der Schnittstelle, die es erfordert, dass der Nutzer wiederholt bestimmte neuronale Signalmuster erzeugt [44, S. 74].^164^ Sie sollen Gehirnfunktionen oder -aktivitäten vielmehr messen, um z. B. Computer oder Smartphones „mit Gedanken“ zu steuern.^165^

 Spätestens wenn der Markt nichtmedizinischer Neuroimplantate in den nächsten Jahrzehnten breite Teile der Bevölkerung erreicht,^166^ sind der nationale und der europäische Normgeber dringend dazu aufgerufen, die regulatorische Lücke zu schließen [44, S. 74 f.; 128, S. 231 ff.] und die normative Architektur auf kommende technologische Innovationen auszurichten.^167^

##### *b) Allgemeine Anforderungen der MPVO an die IT-Sicherheit von Medizinprodukten und unverbindliche Leitlinien*

Anders als etwa Arzneimittel unterliegen Medizinprodukte keiner allgemeinen Zulassungspflicht als Sicherheitsmaßnahme.^168^ Die Hersteller sind vielmehr in eigener Verantwortung gehalten, die einschlägigen Sicherheits- und Leistungsanforderungen zu erfüllen. Dazu gehören auch Anforderungen an die IT-Sicherheit der Systeme (Art. 5 Abs. 1, Abs. 2 i. V. m. Anhang I Nr. 1 und Nr. 17 MPVO) [198, S. 701; 199]. Die Verordnung trägt ihnen aber auf, ein Konformitätsverfahren (Art. 52 MPVO) durchzuführen, um sicherzustellen, dass die Medizinprodukte den Sicherheits‑, Leistungs- und sonstigen rechtlichen Anforderungen entsprechen. In dessen Rahmen hat ggf. auch eine klinische Bewertung (Art. 61 MPVO) zu erfolgen.^169^ Bei dem Konformitätsverfahren wirken sog. *Benannte Stellen* mit, die von staatlichen Stellen akkreditiert und vom Hersteller beauftragt sein müssen [199, S. 531 f.].^170^ Sie führen Audits durch und bewerten das implementierte Qualitätsmanagementsystem, insbesondere dessen Umsetzung und die technische Dokumentation.^171^ Bei Implantaten ist zudem die Koordinierungsgruppe Medizinprodukte zu beteiligen („*Scrutiny Verfahren*“, Art. 54 MPVO) [199, S. 532].

Für die Besonderheiten vernetzter Geräte ist das Medizinprodukterecht indes noch nicht vollständig gewappnet: Die Sicherheits- und Leistungsanforderungen für Medizinprodukte beziehen sich vor allem auf die *Produktsicherheit* (*Safety*) – nicht (unmittelbar) auf die Sicherheit des Geräts als *IT-System* (*Security*) [70, S. 16 ff.].^172^ Dieser Missstand ist ein Relikt aus jener Zeit, als Medizinprodukte über kaum bis keine Softwarekomponenten verfügten und nicht vernetzt waren [70, S. 16 f.].

Anforderungen an die Cybersicherheit für Medizinprodukte mit digitaler Komponente gibt die MPVO lediglich durch allgemeine Bestimmungen vor: Neben der Wiederholbarkeit, Zuverlässigkeit und Leistung (Anhang I Nr. 17.1. MPVO) nach Maßgabe der bestimmungsgemäßen Verwendung des Produkts muss der Produzent es „entsprechend dem Stand der Technik entwickelt und hergestellt“ haben. Dabei sind „die Grundsätze des Software-Lebenszyklus, des Risikomanagements einschließlich der Informationssicherheit, der Verifizierung und der Validierung zu berücksichtigen“ (Anhang I Nr. 17.2. MPVO).^173^ Der Stand der Technik schließt hinreichend sichere Schutzmaßnahmen gegen Sicherheitsangriffe auf sensible Bausteine ein.

Konkretere Leitlinien zu der Frage, wie ein Hersteller die Anforderungen des Anhangs I der MPVO an Cybersicherheit erfüllen kann, gibt die *Guidance on Cybersecurity for Medical Devices* der Koordinierungsgruppe Medizinprodukte vor.^174^ Sie empfiehlt u. a. Mindestanforderungen zur Gewährleistung der IT-Sicherheit – mit einem klaren Augenmerk auf Cybersicherheitsrisiken, die sich auf die Patientensicherheit auswirken können [71, S. 9 f.].^175^ Rechtsverbindlich sind diese Leitlinien indes nicht [201, Rn. 82 f.].^176^ Sie fungieren vielmehr als Auslegungshilfe für die allgemein gehaltenen Sicherheitsanforderungen der MPVO.

Die Leitlinie gibt den Herstellern von Medizinprodukten insbesondere vor, eine *Defense-in-Depth*-Strategie zu entwerfen, die den gesamten Produktlebenszyklus abdeckt [71, S. 15]: Sie soll u. a. Maßnahmen zum sicheren Design und Einsatz, zur Überprüfung und Validierung,^177^ zum Sicherheitsmanagement sowie Spezifikationen der Sicherheitsanforderungen enthalten [71, S. 14 ff.].^178^

Der Anhang der *Guidance* hält eine Liste mit Maßnahmen und Sicherheitsfunktionen vor, die IT-Sicherheit gewährleisten sollen, z. B. Verschlüsselung, persönliche Authentifizierung, automatisches Abmelden, Hardening („Härten“, also Eliminieren nicht benötigter Funktionen, um die Angriffsfläche zu verkleinern [171, S. 146 ff.; 203, S. 248; 204]) und Programme, die vor Schadsoftware schützen oder diese erkennen [71, S. 18, 21 f.].^179^

Darüber hinaus weist die *Guidance* aus gutem Grund an, Zugriffsrechte restriktiv zu vergeben und sichere Authentifizierungsmöglichkeiten vorzuhalten [71, S. 11, 14 f., 21, 37]. So offensichtlich dies klingt, ist es in der bisherigen Praxis doch keine Selbstverständlichkeit. Manche auf dem Markt vertriebenen Medizinprodukte verfügen über keine oder eine unsichere Methode der Authentifizierung [39, S. 264; 64, S. 424 f.],^180^ z. B. Standardpasswörter^181^ oder im Klartext auf dem Gerät gespeicherte Passwörter [104, S. 403]. Es kann außerdem sinnvoll sein, den Zugriff auf ein vernetztes Medizinprodukt auf eine spezifische Raumdistanz zu beschränken, um Fernzugriffe auszuschließen [92, S. 9]. Alternativ zu einer Internetverbindung ist es etwa möglich, die Gehirn-Computer-Schnittstelle z. B. via Bluetooth mit einem anderen System zu koppeln, um die Sicherheit zu erhöhen.^182^

Zudem gibt die *Guidance* den Herstellern auf, im Rahmen eines Verfahrens zum Sicherheitsrisikomanagement das Risiko (insbesondere vorhersehbare^183^ Schwachstellen) zu evaluieren und mögliche Maßnahmen zu dessen Kontrolle und ggf. verbleibenden Restrisiken in Betracht zu ziehen [71, S. 11, 16 f.]. Denn dann fällt es den Herstellern im „Ernstfall“ leichter, einem geregelten Verfahren zu folgen, bei dem Zuständigkeiten und Abläufe vorab geklärt sind [51, S. 153 ff.; 206, S. 2581 f.]. Um langfristige Sicherheit zu gewährleisten, haben Hersteller ihre Medizinprodukte zudem fortlaufend zu testen [71, S. 22 f.].

##### *c) Pflichten nach Inverkehrbringen*

Viele Sicherheitslücken gängiger IT-Systeme treten erst nach einiger Zeit in Erscheinung; fortwährend entstehen bzw. zeigen sich neue Angriffsvektoren [71, S. 23]. Der Hersteller muss daher den gesamten Lebenszyklus des Medizinprodukts, insbesondere der Softwarekomponenten, auch im Blick behalten, nachdem er es in den Verkehr gebracht hat, um das Restrisiko zu begrenzen [207]. Für stark softwaregesteuerte und ggf. lernfähige Systeme [208, S. 19 ff.], wie Gehirn-Computer-Schnittstellen, ist dies mit Blick auf die vielen Angriffsvektoren und -szenarien unentbehrlich.^184^ Die Hersteller müssen ihre Medizinprodukte überwachen,^185^ im Ernstfall die zuständigen Behörden informieren und Präventiv- oder Korrekturmaßnahmen ergreifen (Art. 10 Abs. 12 UAbs. 1, Art. 83 Abs. 4 MPVO). Im Einzelfall können sie auch dazu angehalten sein, ein fehlerhaftes Produkt vom Markt zu nehmen oder zurückzurufen (Art. 95 Abs. 1 MPVO).

##### aa) Schwerwiegendes Vorkommnis

Im Falle sog. schwerwiegender Vorkommnisse muss der Hersteller Sicherheitskorrekturmaßnahmen im Feld vornehmen, um Schäden abzuwenden und Risiken für Patienten zu verringern (Art. 2 Nr. 68, Art. 83 Abs. 4 MPVO) [67, S. 29 f.; 207, S. 301].^186^ Dafür kann er bspw. Sicherheitsupdates aufspielen bzw. zur Verfügung stellen.^187^ Bei schwerwiegenden Vorkommnissen, die auf Sicherheitslücken der IT-Sicherheit beruhen, ist das BSI zu beteiligen (§ 85 Abs. 5 Nr. 1 Medizinprodukte-Durchführungsgesetz). Darüber hinaus treffen den Hersteller – ähnlich wie den datenschutzrechtlich Verantwortlichen (Art. 33 und 34 DSGVO) – Meldepflichten gegenüber den zuständigen Behörden (sog. *Vigilanz*, Art. 87 MPVO).

Ein „schwerwiegendes Vorkommnis“ ist eingetreten, wenn eine Fehlfunktion oder Verschlechterung des Medizinprodukts zur Folge haben kann, dass eine Person stirbt, sich ihr Gesundheitszustand vorübergehend oder dauerhaft schwerwiegend verschlechtert oder dass eine schwerwiegende Gefahr für die öffentliche Gesundheit eintritt (Art. 2 Nr. 65 MPVO). *Nicht schwerwiegend* sind demgegenüber Sicherheitslücken, die es ermöglichen, die Kommunikation eines Medizinprodukts mit einem anderen Gerät^188^ abzuhören oder auf dem Medizinprodukt gespeicherte Daten^189^ auszuspähen oder zu exportieren. Dies gilt gleichfalls für Konstellationen, in denen die Gesundheit des Patienten nur geringfügig, z. B. durch eine verspätete Behandlung oder langsamere Ausführung, beeinträchtigt ist.^190^

Für Gehirn-Computer-Schnittstellen sind schwerwiegende Vorkommnisse deshalb a priori nur bei Varianten denkbar, die das Gehirn exzessiv stimulieren und schwer schädigen können.^191^ Sind dagegen nur leichtere Manipulationen ohne tödliche oder schwerwiegende gesundheitliche Folgen zu gewärtigen, entsteht selbst dann keine Vigilanz-Pflicht, wenn der Patient selbst im Einzelfall die Konsequenzen – vom Vertrauensverlust und Ängsten bis zu leichten Schäden und Schmerzen – als gravierend empfindet.

##### bb) Einfaches Vorkommnis

Auch bei „einfachen“ Vorkommnissen, die unter der Schwelle des Art. 2 Nr. 65 MPVO bleiben, ist der Hersteller nicht von seiner Verantwortung entbunden, für ein Mindestmaß an Sicherheit zu sorgen. Er bleibt verpflichtet, die Konformität des Produkts im Störungsfall (wieder)herzustellen und Korrekturmaßnahmen vorzunehmen (Art. 10 Abs. 12; Art 83 Abs. 4 MPVO). Er muss die Ursache eines potenziellen oder vorhandenen Konformitätsmangels (oder einer sonstigen unerwünschten Situation, vgl. Art. 2 Nr. 67 MPVO) beseitigen.

Aufsichtsrechtliche Maßnahmen ergreifen die zuständigen Behörden in solchen Fällen nur, wenn Medizinprodukte mutmaßlich ein unvertretbares Risiko auslösen oder aus anderen Gründen nicht rechtskonform sind (Art. 94 MPVO). Dann bewerten sie das Produkt in Hinblick auf die Anforderungen der MPVO und fordern den Hersteller z. B. zu Korrekturmaßnahmen auf.^192^ Allerdings muss hierfür ein unvertretbares Gesundheits- und Sicherheitsrisiko für Patienten, Anwender oder andere Personen eintreten (vgl. Art. 95 Abs. 1 MPVO). Insbesondere Schwachstellen, die die Vertraulichkeit von Daten betreffen, erreichen diese kritische Schwelle regelmäßig nicht.^193^

##### *d) Sicherheit durch Datenbanken und Register?*

Zu dem Pflichtenheft der Überwachung und Vigilanz gesellt sich im unionsrechtlichen Regulierungsregime eine europäische Datenbank für Medizinprodukte (*Eudamed*, Art. 33 MPVO; aa) und ein Implantateregister hinzu (§ 1 Abs. 2 Nr. 4 IRegG; bb).

##### aa) Eudamed

*Eudamed* hält Informationen über alle Medizinprodukte vor. Die Datenbank ist u. a. mit dem elektronischen System für Vigilanz und für die Überwachung nach dem Inverkehrbringen verknüpft (Art. 33 Abs. 2 lit. f i. V. m. Art. 92 MPVO).^194^ Sie speichert u. a. Vigilanz- und klinische Prüfungsdaten sowie wie Informationen über die Hersteller.^195^ Ihre Daten sind aber nicht öffentlich zugänglich: Nur die EU-Kommission und die Mitgliedstaaten^196^ dürfen auf sie zugreifen (Art. 33 Abs. 5 MMPVO), um auf Sicherheitsrisiken rasch reagieren zu können.^197^

##### bb) Implantateregister

Anders als Eudamed erfasst das Implantateregister (künftig^198^) auch Informationen über Patienten mit spezifischen Implantattypen, u. a. Cochleaimplantate^199^ und Neurostimulatoren (§ 2 Nr. 1 i. V. m. Anlage IRegG).^200^ Der Unionsgesetzgeber will dadurch Implantatrisiken abwehren sowie die Gesundheit und Sicherheit der Patienten schützen (§ 1 Abs. 2 Nr. 1 IRegG). Deshalb muss die Gesundheitseinrichtung, die für die implantatbezogene Maßnahme verantwortlich zeichnet, in Zukunft u. a. technisch-organisatorische Daten zum Versorgungsprozess, implantatrelevante Befunde sowie individuelle Parameter zum Implantat melden (§ 16 Abs. 1 IRegG). Darunter fallen etwa sicherheitsbezogene Änderungen eines Implantats oder Sicherheitsupdates (§ 2 Nr. 4 IRegG). Mithilfe des Registers können verantwortliche Gesundheitseinrichtungen und Hersteller Patienten ausfindig machen, die unsichere Geräte nutzen,^201^ und sicherstellen, dass sie verfügbare Updates aufspielen (§ 4 Abs. 4, § 29 Abs. 1 Nr. 1, Nr. 3 lit. c IRegG).

Ob sich das neue Implantateregister als geeignetes Instrument entpuppt, um Reaktionen auf schwerwiegende Sicherheitslücken in Implantaten zu koordinieren und die Gefahren für Patienten abzuwehren, steht derzeit aber noch in den Sternen. Als Crux könnten sich zwei Aspekte erweisen: Bislang ist es Patienten zum einen verwehrt, selbst Probleme mit ihren Implantaten zu melden; weder sie noch ihre Ärzte haben Zugang zu den Daten des Registers [209, S. 82; 210, S. 13].^202^ Zum anderen beschränkt sich der Schutz – ähnlich wie die Überwachung nach Inverkehrbringen und die Vigilanz – auf die Gesundheit und (körperliche) Sicherheit der Patienten. Wenn Sicherheitslücken „nur“ eine Einsicht in Daten, die das Implantat erhebt oder verarbeitet, ermöglichen, knüpft sich daran keine Meldepflicht des Herstellers. Dies ist allenfalls dann der Fall, wenn der Hersteller oder die verantwortliche Gesundheitseinrichtung zugleich datenschutzrechtliche Verantwortliche sind (Art. 33, 34 DSGVO).^203^

#### Das (allgemeine) Recht der Cybersicherheit als Lückenschließer?

Jenseits des Rechts der Medizinprodukte und des Datenschutzrechts hält die bestehende Rechtsordnung nur wenige Pflichten vor, welche die IT-Sicherheit in den Verkehr gebrachter Gehirn-Computer-Schnittstelle adressieren.

##### *a) Recht der Sicherheit in der Informationstechnik*

Die Anforderungen an Betreiber Kritischer Infrastrukturen, die das Gesetz über das Bundesamt für Sicherheit in der Informationstechnik (BSIG) und die Verordnung zur Bestimmung Kritischer Infrastrukturen (BSI-KritisV) statuieren,^204^ finden auf Gehirn-Computer-Schnittstellen keine Anwendung. Denn bei der Technologie handelt es sich nicht um unmittelbar lebenserhaltende Medizinprodukte. Sie gehört daher nicht zu den Kritischen Infrastrukturen.^205^ Selbst wenn die Anbieter einer Schnittstelle auf *Cloud-Computing*-Dienste zurückgreifen, um Daten zu speichern oder zu verarbeiten, werden sie nicht selbst zu Anbietern digitaler Dienste (§ 2 Nr. 11 BSIG):^206^ Lediglich Anbieter der Rechenressourcen und Betreiber der Serverfarmen [212, S. 618] müssen Maßnahmen treffen, um die Risiken für die Sicherheit der Netz- und Informationssysteme zu bewältigen, die sie benutzen (§ 8c Abs. 1 BSIG).^207^

Allerdings darf das BSI informationstechnische Produkte und Systeme untersuchen, die auf dem Markt bereitgestellt sind oder werden sollen (§ 7a Abs. 1 BSIG). Von seiner Kompetenz hat das BSI bereits in Kooperation mit mehreren Herstellern Gebrauch gemacht und zahlreiche Medizinprodukte geprüft: Bei jedem Produkt konnte es dabei Sicherheitsmängel feststellen.^208^

##### *b) Produktrecht*

Anforderungen für *nichtmedizinische* Gehirn-Computer-Schnittstellen ergeben sich allenfalls aus dem Produktsicherheits-209 und Produkthaftungsrecht.^210^ Denn Sicherheitslücken können durchaus Produktfehler i. S. d. § 1 ProdHaftG sein [4, S. 195]. Wann insoweit die kritische Schwelle überschritten ist, dekretiert der unbestimmte Rechtsgriff „Stand der Technik“.^211^ Ihn konkretisieren Empfehlungen, Leitlinien^212^ und technischen Standards (z. B. DIN- oder ISO-Normen) [215, S. 522].

Allerdings schützt das Produktrecht nur das Eigentum sowie Körper, Gesundheit und Leben – nicht jedoch Persönlichkeits- und Vermögensinteressen [216, Rn. 2; 217, Rn. 8]. Mit Blick auf die drohenden Beeinträchtigungen der Datensicherheit und Privatheit, die von Cyberangriffen ausgehen, wäre das aber erforderlich. Zudem sind Sicherheitslücken in der Regel beim Inverkehrbringen nicht vorhersehbar, sondern werden erst im Nachhinein erkennbar (§ 1 Abs. 2 Nr. 2, Nr. 5 ProdHaftG).^213^

##### *c) Verbrauchervertragsrecht*

Eine ausdrückliche Pflicht, digitale Produkte durch Updates aktuell und sicher zu halten, kannte das deutsche Recht lange Zeit nicht [218, 220]. Mit Art. 7 Abs. 3 der neuen Warenkauf-Richtlinie^214^ hält eine solche jedenfalls aber für Verbraucherprodukte in die Rechtsordnung Einzug. Den Weg in das nationale Recht ebnet § 475b Abs. 4 BGB [221, S. 1707; 222, S. 315 ff.; 223, S. 455 f.; 224, S. 2890 f.].^215^ Auch die Vertragsmäßigkeit einer gekauften Ware soll sich künftig – je nach den Umständen des Einzelfalls – auf Anforderungen an Datenminimierung, Datenschutz durch Technik und datenschutzfreundliche Voreinstellungen erstrecken.^216^ So ist bspw. ein Verschlüsselungsprogramm vertragswidrig, wenn es konzeptionell nicht geeignet ist, einen unbefugten Zugriff auf die Daten zu verhindern.^217^ Ausschlaggebend ist, was ein Verbraucher vernünftigerweise erwarten kann.^218^ Die Anforderungen, an denen sich digitale Produkte messen lassen müssen, harren indes einer Konkretisierung [223, S. 454].

##### *d) Ausblick auf die unionale Verordnung zur Regulierung Künstlicher Intelligenz*

Für Gehirn-Computer-Schnittstellen wird in absehbarer Zeit die Verordnung für Künstliche Intelligenz (KI-VO)^219^, welche die EU-Kommission kürzlich als Entwurf vorgelegt hat, normative Rahmenbedingungen vorgeben. Gehirn-Computer-Schnittstellen unterfallen dem Begriff „Künstliche Intelligenz“.^220^ Denn sie verwenden verschiedene statistische Methoden und maschinelles Lernen, um Gehirnsignale zu klassifizieren.^221^ Gehirn-Computer-Schnittstellen, die der MPVO unterfallen,^222^ stuft der Verordnungsentwurf als Hochrisiko-KI ein, sofern ihre Klassifizierungsalgorithmen Sicherheitskomponenten sind.^223^ Darunter fallen etwa Neurostimulatoren, die ihren Nutzer schädigen können, wenn sie fälschlicherweise elektrische Impulse abgeben oder ausfallen und z. B. epileptische Anfälle nicht verhindern oder lindern. Hochrisiko-KI spannt der Gesetzesvorschlag in ein engmaschiges Regulierungskorsett: Die Anbieter müssen die Genauigkeit, Robustheit und Cybersicherheit des KI-Systems gewährleisten^224^ sowie ein Risiko‑, Qualitäts- und Überwachungsmanagement einführen.^225^ Insbesondere müssen solche Systeme gegen Versuche Dritter, Systemschwachstellen auszunutzen, widerstandsfähig sein.^226^ Während des Betriebs sollen die KI-Systeme Ereignisse in Logs festhalten, damit sie nachvollziehbar und überprüfbar sind.^227^ Werden Gehirn-Computer-Schnittstellen zur biometrischen Identifikation oder Emotionserkennung eingesetzt, sind die Nutzer hierüber zu informieren (Art. 52 (2) KI-VO).

#### Das Recht der Cybersicherheit von Gehirn-Computer-Schnittstellen als lückenhaftes Regulierungsmosaik

Das Medizinprodukterecht reguliert die Cybersicherheit von Gehirn-Computer-Schnittstellen bisher nur fragmentarisch. Es erfasst einerseits keine Verbraucher- bzw. nicht medizinische Geräte – und zwar selbst dann nicht, wenn ein solches Gerät (in der Zukunft) unmittelbar in den menschlichen Schädel implantiert wird; damit teilen Gehirn-Computer-Schnittstellen das Schicksal vieler anderer Geräte im „Internet der Dinge“.^228^ Zum anderen fallen Cyberangriffe auf Medizinprodukte, die „nur“ die Privatsphäre des Nutzers verletzen, nicht in das Regelungsregime der Vigilanz und unterliegen daher nicht der Meldepflicht.

Das Datenschutzrecht schließt diese Lücke nicht. Denn es hält nur Pflichten für die personenbezogene Datenverarbeitung durch den Verantwortlichen und den Auftragsverarbeiter, also die Prozesse einer Software, bereit. Den Hersteller der Hardware adressiert die DSGVO demgegenüber nicht unmittelbar. Das neue Verbrauchervertragsrecht erhöht aber im Gefolge der neuen Warenkauf-Richtlinie die IT-Sicherheit von Verbraucherprodukten: Sie knüpft die Vertragsmäßigkeit eines Produkts auch an dessen IT-Sicherheit und etabliert Aktualisierungspflichten.

## Vorschläge für ein Regelungsregime der Cybersicherheit von Gehirn-Computer-Schnittstellen

Heute mag Neuroenhancement noch wie ein Subkulturphänomen wirken. In den nächsten Jahren werden sich jedoch mehr und mehr Menschen seinen vielfältigen technischen Möglichkeiten öffnen. Damit können weitreichende und bislang nicht absehbare Auswirkungen auf die Gesellschaft sowie die Menschheit als Ganzes einhergehen. Um der Gefahren Herr zu werden, die von Angriffen auf Gehirn-Computer-Schnittstellen ausgehen, ist der Staat aufgerufen, normative Lücken zu schließen und Vorsorgemaßnahmen zu ergreifen. Ansatzpunkte können nicht nur neue Vorschriften für Hersteller und Betreiber (I.), sondern auch eine Intensivierung bzw. Effektivierung der behördlichen Aufsicht (II.) sowie Haftungs- und Aufklärungspflichten für Hersteller, Betreiber und medizinisch-technisches Personal (III.) sein.

### Regulierung von Gehirn-Computer-Schnittstellen

#### Ausweitung des Medizinprodukterechts

Bislang erfasst die Rechtsordnung Neuroimplantate nur bei medizinischer Zweckbestimmung (Art. 1 Abs. 2 i. V. m. Anhang XVI MPVO).^229^ Um den Sicherheitsrisiken implantierter Gehirn-Computer-Schnittstellen schlagkräftig zu begegnen, sollte der Unionsgesetzgeber den normativen Radius der MPVO pro futuro auf Implantate zu Enhancement-Zwecken ausweiten.^230^ Er könnte entweder konkrete Ausgestaltungen wie „Neuroimplantate“ oder den abstrakten Zweck des *Enhancements* in die Liste des Anhangs XVI MPVO aufnehmen. Konkrete Produktarten in die Liste aufzunehmen, hat den Nachteil, dass die Liste der dynamischen technischen Entwicklung ständig hinterherhinkt. „*Cognitive Enhancement*“ als normativer Anknüpfungspunkt hat demgegenüber den Charme auch künftige, noch nicht bekannte Technologien zu erfassen und mitzuregulieren [44, S. 81]. Dadurch fallen dann aber auch jegliche EEG-Headsets in den Anwendungsbereich der MPVO. Nichtmedizinische EEG-Headsets gehen in ihrer Invasivität typischerweise über *Wearables* wie Fitness-Tracker, Smartwatches oder Datenbrillen nicht hinaus: Sie erheben außerhalb des Körpers Daten, auch über physiologische Vorgänge (z. B. messen sie die Herzfrequenz oder – im Fall von EEG-Headsets – Gehirnwellen) [225]. Alle EEG-Headsets der MPVO zu unterwerfen, ist daher nicht angezeigt. Es mag zwar seltsam anmuten, die gleichen EEG-Systeme, die mal medizinischen, mal „privaten“ Zwecken dienen, unterschiedlichen Standards zu unterwerfen [44, S. 77]. Allerdings stellen die höheren Anforderungen an Medizinprodukte neben der Sicherheit auch die Leistung, insbesondere den klinischen Nutzen, sicher.^231^ Das Kardinalproblem privat genutzter EEG-Headsets liegt dagegen schwerpunktmäßig im Datenschutz und der datenschutzkonformen Ausgestaltung des Produkts [226, S. 9; 227].

Mit Blick auf diese Risiken empfiehlt es sich, den Begriff des Medizinprodukts so zu verändern, dass er gleichermaßen *Enhancement* umfasst, z. B. indem die MPVO auf die Interaktion des Produkts mit dem Körper oder auf die Risiken für den Nutzer abstellt [44, S. 80 ff.]. Hierdurch schlüpfen jegliche stimulierende und implantierte Gehirn-Computer-Schnittstellen unter den Schirm des Medizinprodukterechts, ohne rein messende, nicht invasive EEG-Geräte miteinzubeziehen.

#### Security by Design

Über den Anwendungsradius der MPVO hinaus sollte der Normgeber auch in materieller Hinsicht nachjustieren – sowohl an den Anforderungen an Produkte sowie an den Herstellerpflichten.^232^

Die bisher erarbeiteten, feinteiligen Leitlinien der Koordinierungsgruppe Medizinprodukte zur Gewährleistung der Cybersicherheit von Medizinprodukten erfüllen zwar eine wichtige Orientierungsfunktion. Ihre Steuerungswirkung leidet aber unter ihrem Empfehlungscharakter: Die Hersteller sind nicht verpflichtet, ihnen Folge zu leisten.^233^ Die wichtigsten Mindestanforderungen (z. B. Verschlüsselung, sichere Authentifizierung und Angriffserkennungssoftware) sollte die Richtlinie in den Anhang I (Nr. 17) aufnehmen und ihnen hierdurch normative Bindungswirkung verleihen.^234^ Bspw. sollte sie als Grundsatz ausdrücklich und verbindlich festlegen, dass Medizinprodukte ruhende Daten verschlüsseln *müssen* und nur verschlüsselt mit anderen Geräten kommunizieren dürfen.^235^

Im Rahmen der MPVO sollte die Koordinierungsgruppe Medizinprodukte weitergehende Anforderungen an die Technikgestaltung formulieren und – ggf. in Abstimmung mit Datenschutzbeauftragten und Cybersicherheitsbehörden^236^ – Basismaßstäbe für *Security by Design* festlegen. Dieser Katalog muss sich dynamisch an aktuelle Bedrohungen anpassen. Bspw. reicht es dann nicht mehr, nur den Benutzerzugriff sicher zu gestalten. Wenn sich Medizinprodukte automatisiert aktualisieren, sollten sie zudem verifizieren, dass die Updates tatsächlich vom Hersteller stammen und unverfälscht sind [45, S. 10 f.].^237^

Für Apps, die via Gehirn-Computer-Schnittstelle (oder auf andere Weise) Gesundheitsdaten verarbeiten, ließen sich integrierte Filter vorschreiben, die nur bestimmte Gehirnsignale zur Auswertung gelangen lassen. Medizinprodukte können dann nur solche Daten verarbeiten und speichern, die erforderlich sind, um ihre Funktion zu erfüllen – und gerade nicht das gesamte Spektrum der gemessenen neuronalen Aktivitäten. Denkbar ist auch die Vorgabe, Gehirnsignale (soweit sie nicht für den jeweiligen Einsatzzweck zwingend erforderlich sind) erst dann auswerten zu dürfen, nachdem sie einen Anonymisierungsmechanismus durchlaufen haben.^238^ Zusätzlich könnte der Gesetzgeber Betreiber von App-Plattformen für Gehirn-Computer-Schnittstellen dazu verpflichten, die dort angebotenen Anwendungen auf ihre Sicherheit und Vertrauenswürdigkeit hin zu überprüfen, bevor sie zum Download freistehen [14, S. 37].

Als Ausdruck eines digitalen Verbraucher- und Patientenschutzes sollten die Aufklärungs- und Informationspflichten der Hersteller und Ärzte [4, S. 194] verstärkt die Risiken und Besonderheiten der IT-Sicherheit einbeziehen, namentlich zusätzlich zu den besonderen Aufklärungspflichten über die gesundheitlichen Risiken [4, S. 194 f.] auch zur Aufklärung über die IT-Sicherheit verpflichten. Wer eine Schnittstelle nutzt, sollte z. B. einen Hinweis erhalten, was bei ihrer Kopplung mit einem Smartphone zu beachten ist.

#### Konsequenzen von Vorkommnissen

Bislang ziehen nur schwerwiegende Vorkommnisse mit gravierenden Gefahren für die Patientensicherheit strenge Vigilanz-Pflichten der Hersteller nach sich. Auch unterhalb dieser Schwelle können Medizinprodukte, insbesondere Neurostimulatoren,^239^ im Fall eines Cyberangriffs aber gefährliche Folgen zeitigen.^240^ Statt es den Herstellern zu überlassen, solche Sicherheitslücken zu beheben, sollten sie auch hier die zuständigen Behörden über Vorkommnisse und Sicherheitskorrekturen informieren müssen, damit diese das Produkt überprüfen und ggf. Maßnahmen (Art. 94, 95 MPVO) einleiten können.

Insgesamt kommt im Medizinprodukterecht der Schutz der Privatsphäre im Vergleich zum Schutz der Patientensicherheit derzeit zu kurz:^241^ Gefährdet eine Sicherheitslücke die Vertraulichkeit des Medizinprodukts, löst dies nur Korrektur-, nicht aber Meldepflichten des Herstellers aus. Es greifen lediglich die Meldepflichten aus Art. 33 DSGVO gegenüber der datenschutzrechtlichen Aufsichtsbehörde – allerdings nur, sofern der Hersteller einer Gehirn-Computer-Schnittstelle zugleich auch datenschutzrechtlicher Verantwortlicher ist.^242^ Die datenschutzrechtlichen Pflichten und das Medizinprodukterecht verdichten sich mithin nicht zu einem vollständigen Schutzportfolio: In Fällen, in denen der Hersteller nicht Verantwortlicher ist, erfasst weder das Medizinprodukte- noch das Datenschutzrecht die Gewährleistung der Vertraulichkeit. Hier sollte der Normgeber dringend nachbessern. Die MPVO sollte im Einzelnen konkretisieren, welche Pflichten bestehen, wenn die Privatheit und informationelle Selbstbestimmung der Patienten gefährdet sind.

Dass Medizinprodukte zunehmend vernetzt sind und – nicht zuletzt aufgrund des demografischen Wandels – immer stärker zum Einsatz kommen, macht es unerlässlich, auf Sicherheitslücken schnell zu reagieren. Die Leitlinien der Koordinierungsgruppe Medizinprodukte formulieren bereits Vorgaben für die Abläufe, Prozesse und Organisation des Managements von Vorkommnissen. Das Anforderungstableau sollte künftig in Gestalt eines delegierten Rechtsaktes verbindliche Regeln formulieren, bedarf dafür aber zugleich noch weiterer und konkreterer Vorgaben.^243^

Im Anschluss an ein Vorkommnis kann es erforderlich sein, das Medizinprodukt erneut zu prüfen und zu bewerten, jedenfalls aber sollte es verpflichtend sein, den Mangel aufzuarbeiten und forensisch zu analysieren [101, S. 132]. Zur Konkretisierung sollten auch insoweit delegierte Rechtsakte entstehen. Eine Blaupause liefert das Recht der IT-Sicherheit: Betreiber Kritischer Infrastrukturen müssen mindestens alle zwei Jahre einen Nachweis gegenüber dem BSI darüber erbringen, dass sie alle Anforderungen an die IT-Sicherheit erfüllen (§ 8a Abs. 3 BSIG). Das BSI kann prüfen, ob die Betreiber die Vorgaben tatsächlich einhalten (§ 8a Abs. 4 BSIG). Eine ähnliche turnusmäßige Überprüfung bietet sich – jedenfalls für risikoreiche Produktklassen – an. Bislang sieht die MPVO lediglich die Prüfung von Sicherheitsberichten (Art. 86 Abs. 2 MPVO) und Stichproben-Kontrollen vor (Art. 93 Abs. 1 MPVO).

### Behördliche Aufsicht

Neben der Regulierung von Gehirn-Computer-Schnittstellen gilt es, eine wirksame behördliche Aufsicht über die Einhaltung der Vorgaben sicherzustellen. Bislang teilt sich ein kleinteiliges Mosaik unterschiedlicher Behörden die Zuständigkeit auf: neben den Datenschutzbehörden vor allem das Bundesinstitut für Arzneimittel und Medizinprodukte (BfArM) und das BSI. Zusätzlich bestehen sporadische Zuständigkeiten des Bundesamts für Verbraucherschutz und Lebensmittelsicherheit (BVL) und der Marktüberwachungsbehörden.^244^

Die verschiedenen Behörden verfügen jeweils über einen eng begrenzten Kompetenz- und Erfahrungsschatz. Fragmentierte Zuständigkeiten erschweren es, die Risiken von Gehirn-Computer-Schnittstellen ganzheitlich zu erfassen. Schon aus verfassungsrechtlichen Gründen lassen sich die Zuständigkeiten der Behörden aber nicht ohne Weiteres in den Händen einer einzelnen Bundesbehörde bündeln. Empfehlenswert ist es jedenfalls, die Zusammenarbeit der Behörden im Kooperationsgruppen zu optimieren [101, S. 129 ff.].^245^ Z. B. könnten Mitarbeiter des BfArM, die Erfahrung bei der Aufsicht über Medizinprodukte gesammelt haben, gemeinsam mit IT-Sicherheitsexperten des BSI im Tandem Produkte auswählen und überprüfen oder Konzepte zur Fortentwicklung der IT-Sicherheit (ggf. gemeinsam mit den Herstellern) erarbeiten.

Die Exekutive könnte überdies noch stärker eine Schlüsselrolle beim Vorfallsmanagement übernehmen: Sie kann eine zentrale Meldestelle einrichten, die Vorkommnisse – ähnlich der *National Vulnerability Database* in den USA – in Datenbanken aufnimmt und öffentlich zugänglich macht.^246^ Das BSI könnte unmittelbar an der Beseitigung von Störungen mitwirken, Maßnahmen hierzu anordnen oder (auf Ersuchen des Herstellers oder Verantwortlichen) diese Maßnahmen selbst treffen.^247^

## Fazit

Gehirn-Computer-Schnittstellen beflügeln nicht nur die Vision, die kognitiven Möglichkeiten des Menschen zu erweitern, seine Gedanken zu lesen sowie ihn eines Tages gar mit künstlicher Intelligenz zu verschmelzen. Als Kehrseite machen sie es in Zukunft auch technisch möglich, gehirnbasierte Lügendetektoren zu verwenden,^248^ Daten einer Gehirn-Computer-Schnittstelle als Beweismittel heranzuziehen^249^ oder ähnlich einer Telekommunikations- oder Quellentelekommunikationsüberwachung Neuroenhancement-Produkte des Terrorismus Verdächtigter zu überwachen.^250^

Je stärker nicht nur Smartphones, Wearables und Smart-Home-Produkte, sondern auch Neurotechnologien unser Privat- und Intimleben steuern, umso mehr müssen Gehirn-Computer-Schnittstellen nicht nur im physischen Sinne sicher sein, sondern auch ihre Integrität und Vertraulichkeit als IT-Systeme gewährleisten.

Einzelne Anforderungen an die Cybersicherheit von Gehirn-Computer-Schnittstellen formuliert die Rechtsordnung bereits. Ein vollständiges Regelungskonzept, das die Sicherheit der Anwendungen hinreichend verbürgt, lässt sie aber vermissen. Während das Medizinprodukterecht die IT-Sicherheit jedenfalls mit Blick auf die Patientensicherheit reguliert, gilt für *nichtmedizinische* Gehirn-Computer-Schnittstellen das gleiche (niedrige) Schutzniveau, das auch alle anderen vernetzten Geräte im Internet der Dinge erfüllen müssen. Die offenen Regelungslücken bei (nichtmedizinischen) Gehirn-Computer-Schnittstellen legen einen grundlegenden Mangel des gesamten Regelungsgeflechts offen: Der Gesetzgeber versteht Cybersicherheit trotz allgegenwärtiger Vernetzung noch nicht als integralen Bestandteil und notwendige Anforderung an digitale Produkte.^251^

Nur das Datenschutzrecht fängt bislang sicherheitsrelevante Verletzungen der Privatsphäre auf einer allgemeinen Ebene ab. Es ist allerdings nicht auf die spezifischen Gefahren von Gehirn-Computer-Schnittstellen zugeschnitten. Sein Fokus richtet sich seinem Wesen nach nur auf Datenverarbeitungsprozesse, erfasst aber nicht unmittelbar die Anforderungen für sichere datenverarbeitende Produkte, die Hersteller einzuhalten haben.

Die neuen EU-Richtlinien zu digitalen Produkten können die klaffenden Lücken immerhin ein Stück weit schließen. Der Unternehmer ist verpflichtet, Software-Updates bereitzustellen, um die Vertragsmäßigkeit aufrechtzuerhalten. Auch der Verordnungsentwurf der EU-Kommission zur KI-Regulierung sieht strenge Anforderungen an jene Sicherheitskomponenten vor, die auf KI-Systemen basieren.

Hinter den Idealanforderungen bleibt der rechtliche Status quo indes weit zurück – anders als bspw. im Spiel *Cyberpunk 2077*. Dort ist das medizinisch-technische Personal des Konzerns *Trauma Team* perfekt darauf vorbereitet, Menschen zu retten, die physischen Verletzungen oder Cyberangriffen zum Opfer gefallen sind: Implantate wählen bei einer Fehlfunktion eigenständig den Notruf und *Trauma Team* repariert das Gerät oder tauscht es in Windeseile aus – sofern hierfür der erforderliche (und teure) Versicherungsschutz besteht.^252^ Eine Vollkaskoabsicherung für Gehirn-Computer-Schnittstellen wird es in der Realwelt der Zukunft nicht geben. Für ein adäquates Sicherheitsniveau sollte der Gesetzgeber aber zeitnah sorgen. Sonst erfasst das vielbeschworene Internet der Unsicherheiten [236] nicht nur das Internet der Dinge, sondern auch unsere Körper – und Gehirne.

## Footnotes

1 Im Englischen: *Brain-Computer Interfaces* [1]. Auch die Begriffe *Brain-Machine Interfaces* [2] oder *Neural Interface Systems* [3] sind verbreitet. Eine juristische Betrachtung findet sich bei Heene [4]. Für anschauliche und unterhaltsame Einführungen zum Thema, siehe Urban [5], aber auch Schmidt und Jähn [6] sowie maiLab [7].

2 Cyberpunk 2077, CD Projekt RED (2020).

3 https://cyberpunk.fandom.com/wiki/Cyberdeck#Cyberpunk_2077.

4 Dabei übernimmt der Täter die Kontrolle über das Opfer. Dogmatisch betrachtet läge daher kein Selbstmord, sondern ein Mord in mittelbarer Täterschaft vor (§§ 211, 25 Abs. 1 Alt. 2 StGB). Eine Liste aller Quickhacks im Spiel findet sich unter https://cyberpunk2077.wiki.fextralife.com/Quickhacks.

5 Bei ihnen handelt es sich um Personen, deren untere Körperhälfte querschnittsgelähmt ist.

6 Bislang gelingt es nur schleppend und in begrenztem Maße, Prothesen mittels Gehirnsignal zu steuern. Das wohl prominenteste Beispiel ist das einer 58-jährigen Querschnittsgelähmten, die nach fast 15 Jahren Lähmung das erste Mal wieder in der Lage war, einen Schluck Kaffee zu trinken [8].

7 Z. B. Emotiv (https://www.emotiv.com/) oder Neurosky (http://neurosky.com/).

8 Siehe https://quantifiedself.com/. Gerade „*wearable electronics*“ (tragbare Elektronik mit Sensoren), ermöglichen es, „Exosinne“ zu entwickeln: Blutdruckwerte, Puls, Stresslevel, u. v. m. werden durch „self-tracking“ *erfahrbar* [9, S. 95 f.]. Es entstehe, so die These, ein „Exoself“, das sich über den eigenen Körper hinaus gleichermaßen auf Smartphones, Wearables usw. erstreckt: „The individual body becomes a more knowable, calculable, and administrable object through [Quantified Self] activity, and individuals have an increasingly intimate relationship with data as it mediates the experience of reality“ [9, S. 85]. Vgl. zu „Do-it-Yourself“-Neurotechnologie und den verschiedenen dahinter stehenden Motivationen: „Viewed broadly, the home use of brain stimulation sits at the nexus of maker and DIY cultures, citizen science movements, and self-experimentation and self-tracking initiatives“ [10, S. 4].

9 Siehe https://www.emotiv.com/workplace-wellness-safety-and-productivity-mn8.

10 Neurosky bietet bereits EEG-Gaming-Headsets und Apps an; https://store.neurosky.com/collections/apps.

11 Steuern Beschäftigte mithilfe von Gehirn-Computer-Schnittstellen Exoskelette, stellen sich Fragen des Arbeitsschutzes und Beschäftigtendatenschutzes [13, S. 626 ff., 635 f.].

12 Z. B. von Emotiv, https://www.emotiv.com/workplace-wellness-safety-and-productivity-mn8/. Emotionale Reaktionen können anzeigen, ob eine Person zufrieden und entspannt oder genervt, gestresst bzw. irritiert ist. Auf dieser Basis könnten Apps z. B. Meditationsprogramme oder die Musikauswahl anpassen [14, S. 36].

13 Die Firma Nemos vertreibt z. B. ein Neurostimulator in unauffälliger Earbud-Form [16].

14 Aus diesem Kontext stammt auch die erste Verwendung des Begriffs „Cyborg“ – ein Akronym für *Cybernetic Organism*. Es handelt sich um einen (biologischen) Organismus, der mit steuerbaren Maschinen(teilen) ausgestattet ist (Kybernetik) [22, S. 30 f.].

15 So verlautbarte *Elon Musk* auf Twitter, das Unternehmensmotto von Neuralink sei „*If you can’t beat em, join em*“: Wenn man Künstliche Intelligenz nicht übertreffen kann, solle man sich einfach mit ihr verbinden, Twitter vom 09.07.2020, https://twitter.com/elonmusk/status/1281121339584114691. Inwieweit diese Strategie realisierbar ist, ist technisch und philosophisch umstritten. Eine kritische Diskussion der Verbindung organischer mit synthetischer Intelligenz findet sich u. a. bei Schneider [25, S. 72 ff., 191 f.].

16 In diesem Zusammenhang setzt Kurzweil [27, S. 316 f.] auf Nanotechnologie. Kritisch: Gabriel [28, S. 18 ff.], Schneider [25, S. 72 ff.] und Žižek [29, S. 135 ff., 176 f.].

17 In diesem Fall firmiert die Gehirn-Computer-Schnittstelle auch unter dem Begriff „Neuroprothese“. Zur Steuerung eines Exoskeletts durch Gehirn-Computer-Schnittstellen Martini/Botta [13, S. 626].

18 Da die Physiologie und die neuronalen Signalstrukturen bei jedem Menschen unterschiedlich sind, muss das System für jeden Nutzer eine Trainingsphase durchlaufen, um ein personalisiertes Modell für den Klassifizierungsalgorithmus zu erhalten. Zudem muss ggf. im Laufe der Zeit nachjustiert werden, da sich das Gehirn des Nutzers im Rahmen seiner Neuroplastizität stetig verändert [31, S. 782]. Zum Einsatz adaptiver Dekodier-Algorithmen siehe Lebedev und Nicolelis [2, S. 795].

19 Z. B. von Neurosky, https://store.neurosky.com/collections/apps. Siehe zu App-Stores für Gehirn-Computer-Schnittstellen Bonaci, Calo und Chizeck [14, S. 32 ff.].

20 Messungen der Neuronenaktivität oder der Veränderungen des Blutsauerstoffgehalts ermöglichen es, die *funktionellen* Gehirnaktivitäten darzustellen, die mit der Planung und Durchführung bestimmter Aufgaben zusammenhängen [32, S. 202 f.]. Die Qualität der Aufzeichnung neuronaler Aktivitäten schwankt allerdings mitunter sehr stark: v. a. bei EEG-basierten Gehirn-Computer-Schnittstellen treten Rauschsignale auf. Das gründet vor allem darauf, dass die auf der Kopfhaut angebrachten Elektroden verrutschen oder versehentlich elektrische Muskelaktivität messen können [1, S. 267; 2, S. 805].

21 Z. T. wird der Nutzer der Gehirn-Computer-Schnittstellen bestimmten Stimuli ausgesetzt, z. B. bei Lügendetektoren, die ihm Fakten, Zeugenaussagen oder Beweisgegenstände präsentieren (sog. P300-basierende Lügendetektoren) [1, S. 249 ff.]. Zur Lügendetektion durch funktionelle Magnetresonanztomographie (fMRI) siehe Farah et al. [33].

22 Hierbei kommen Methoden des maschinellen Lernens zur Anwendung [1, S. 71 ff.; 34]. Maschinelles Lernen kommt auch zum Einsatz, um die Bewegungen der Neuroprothesen besser zu steuern [35]. Die fehlende Transparenz der Ergebnisfindung wirft ethischen Fragen auf [36].

23 Derzeit entwickelt die Forschung Methoden, um Gehirnaktivitäten vorherzusagen und dadurch Anfälle noch besser zu verhindern [39, S. 256].

24 Siehe z. B. die Neurostimulationssysteme von Medtronic, https://www.medtronic.com/de-de/fachkreise/produkte/neurologie-schmerztherapie/neurostimulationssysteme-zur-tiefen-hirnstimulation.html.

25 Diese Technologie weist Ähnlichkeiten zum *Tactile Internet* auf: Menschen oder Maschinen werden künftig reale oder virtuelle Objekte oder Prozesse aus der Ferne in Echtzeit ansteuern, wahrnehmen und kontrollieren können [42, 43], siehe hierzu allgemein https://ti.committees.comsoc.org/.

26 Zu weiteren nichtinvasiven Methoden: Lebedev/Nicolelis [2, S. 805 ff.]. Es gibt zudem semiinvasive Methoden, bei denen die Elektroden unterhalb des Schädels auf der Hirnoberfläche angebracht sind [1, S. 149 ff.].

27 Die transkranielle Magnetstimulierung erzeugt ein Magnetfeld, das ca. 3 bis 5 cm in den Schädel reicht und dort durch elektromagnetische Induktion Neuronengruppen aktiviert [1, S. 33 f.].

28 Transkranielle Gleichstromstimulation (*transcranial direct current stimulation, tDCS)* verändert Gehirnaktivitäten mittels eines elektrischen (Gleichstrom‑)Impulses, den sie auf der Kopfhaut auslöst. Diese Methode kommt u. a. zur Anwendung, um Depressionen zu behandeln [44, S. 70 ff.].

29 So z. B. bei Musk/Neuralink [46].

30 Allerdings ist es bislang nur möglich, eine Person basierend auf Gehirnsignalen einer anderen Person zu stimulieren [48]. An telepathischer Kommunikation forscht jedoch z. B. die *Defense Advanced Research Projects Agency* (DARPA) der USA im Rahmen des Programms „Silent Talk“ [49]. Zu den ethischen Dimensionen von Mehrpersonen-Brain-to-Brain-Interfaces: Hildt [50].

31 Die Begriffe „IT-Sicherheit“ und „Cybersicherheit“ werden hier als Synonyme verwendet. Angriffsszenarien, die organisationale Schwächen oder den Faktor „Mensch“ ausnutzen (*Social Engineering*), klammert der Beitrag weitgehend aus. Zu diesen Fragen siehe bspw. Meeuwisse [51, S. 73 ff.].

32 Mit Neurosecurity setzt sich bspw. die Agentur für Innovation in der Cybersicherheit auseinander [53].

33 In der EU prüfen akkreditierte Stellen Medizinprodukte (siehe 2.2.2.b), in den USA die Food and Drug Administration (siehe zum Überblick https://www.fda.gov/medical-devices/products-and-medical-procedures).

34 Eine der getesteten Insulinpumpen sollte mit einer PIN gesichert sein, übermittelte diese aber im Klartext. Zudem erfragten nur die mobilen Anwendungen die Nutzer-PIN, weshalb der direkte Zugriff auf die Insulinpumpe möglich blieb. Die Arzt-PIN – die den Zugriff auf das Arztmenü ermöglicht, über das sich z. B. die maximale Insulintagesdosis verändern lässt – war für alle Pumpen gleich [58, S. 44 ff.].

35 Zu diesem Zweck haben die Ärzte die drahtlose Kommunikation mit dem Implantat deaktiviert [62]. Ein implantierter Kardioverter-Defibrillator misst den Herzschlag und gibt bei Irregularitäten einen leichten elektrischen Impuls ab, um den Rhythmus des Herzschlags zu normalisieren.

36 Erwägungen zur nationalen Sicherheit finden sich bei [64, S. 427 ff.].

37 Die möglicherweise erfüllten Straftatbestände diskutieren Gasson und Koops [39, S. 266 ff.]. Ebenso können Cyberangriffe auf (reguläre) IT-Systeme gravierende Folgen für Leben, Körper und Gesundheit zeitigen. So keimten nach einem Ransomware-Angriff auf die Uniklinik Düsseldorf Spekulationen darüber auf, ob dieser den Tod einer Frau wegen einer verspäteten Behandlung verursacht hat [65]. Die Ermittlungen hat die zuständige Staatsanwaltschaft aber eingestellt. Sie ging davon aus, dass die Frau bei rechtzeitiger Behandlung ebenso gestorben wäre [66]. In der Vergangenheit konnten Angreifer blinkende Videos oder GIFs an Epileptiker schicken und so einen Anfall auslösen [67]; einen ähnlichen Angriff hat es schon im Jahr 2008 gegeben [68].

38 Zur parallelen Problemlage bei neurologischen und genetischen Daten: Spranger [69, S. 41 f.].

39 Sog. „*eavesdropping*“ [52, S. 3]. Dieses Problem stellt sich besonders bei Gehirn-Computer-Schnittstellen, die emotionale Zustände erkennen, z. B. um Stress zu vermeiden oder Depressionen zu behandeln.

40 Art. 32 Abs. 1 Hs. 2 lit. b DSGVO sowie [70, S. 1; 71, S. 8 f.]. Hellmann führt noch Authentizität und Non-Repudiation, d. h. die Nichtabstreitbarkeit einer Datenübertragung, auf [72, S. 5 f.].

41 Eine sog. *Jamming Attack* kann durch elektromagnetisches Rauschen die Aufnahme und möglicherweise auch die Stimulation verhindern [45, S. 11].

42 Mithilfe einer *Replay and Spoofing Attack* lassen sich frühere oder synthetische Signale einspielen und echte Signale imitieren [45, S. 11].

43 Da in der Regel komplexe Algorithmen und Lernmodelle zum Einsatz kommen, die auf den Nutzer gleichsam geeicht sind, besteht ein großes Risiko, dass ein Angriff in die Datenverarbeitung „im Hintergrund“ unentdeckt bleibt [74, S. 123 f.]. Maschinelles Lernen macht sog. *Adversarial Attacks* möglich, die Klassifikationsergebnisse manipulieren. Bspw. können *Poisoning Attacks* den Lernprozess so beeinflussen, dass die lernfähige Software einen bestimmten Input fehlerhaft klassifiziert und sich eine Hintertür zur Kontrolle des Outputs öffnet [45, S. 14 f.].

44 Siehe Ienca und Haselager [74, S. 123]; allgemein zu gehirngesteuerten Rollstühlen: Rao [1, S. 241 f.].

45 *Spoofing Attacks* schleusen Schadsoftware in ein System ein, indem die Angreifer diese identisch zu vertrauenswürdiger Software gestalten und z. B. in einem App Store anbieten [14, S. 33; 45, S. 16].

46 Siehe 1.2.2.a) cc).

47 Grundsätzlich gehen von *Deep-Brain-*Stimulatoren nur geringe Risiken aus: Das größte Risiko für Patienten ist (statistisch gesehen) Selbstmord. Allerdings kommen – wenn auch selten – Komplikationen wie Gehirnblutungen, epileptische Anfälle oder Gedächtnisstörungen vor [75].

48 Zur Vertraulichkeit im Datenschutzrecht vgl. Martini [77, Rn. 35d].

49 Jedenfalls spricht sich *Elon Musk* für telepathische Kommunikation aus [79]; kritisch hierzu Dingemanse [80].

50 Zu *Cognitive Function Augmentation*: Denning, Matsuoka und Kohno [52, S. 3]; zur Kontrolle und Manipulation affektiver Zustände: Steinert und Friedrich [38, S. 355, 361 f.]. Pugh et al. weisen darauf hin, dass es denkbar ist, Nutzern ein Verhalten anzutrainieren, in dem eine Schnittstelle gezielt manipulativ positive oder bestrafende Stimuli aussendet [78, S. 221].

51 Siehe bereits 1.1.2.

52 Siehe hierzu aus datenschutzrechtlicher Perspektive 2.2.1.a).

53 Diese Stimuli können offensichtlich oder unterschwellig sein [14, S. 36]; siehe hierzu die Experimente von Martinovic et al. [81].

54 Angriffe mittels Stimuli verlaufen leichter und genauer, wenn die Trainingsphase beendet und die Gehirn-Computer-Schnittstelle sorgfältig kalibriert wurde. Bei Freizeitgeräten werden die Nutzer das regelmäßig selbst durchführen [81, S. 151]. Ein solcher Angriff kann aber nur dann zum Erfolg führen, wenn der Angreifer über einen gewissen Zeitraum hinweg den „Input“ bzw. die dem Nutzer präsentierten Stimuli kontrolliert [81, S. 155].

55 Daten zu physiologischen Prozessen eines Implantats lassen sich künftig als Beweismittel heranziehen. Im US-Bundesstaat Ohio haben die Strafverfolgungsbehörden bspw. einen Täter mit Hilfe der Herzfrequenzdaten seines Herzschrittmachers einer Brandstiftung überführt [88].

56 So lassen sich für Informationen zu Krankenversicherungen Preise mitunter bis zu umgerechnet $ 22 erzielen [90]. Im Jahr 2016 haben Täter die Gesundheitsdaten von mehr als 10 Mio. US-Bürgern für $ 486.000 erbeutet [76, S. 656 f.; 91].

57 Täter könnten Informationen über die Identität oder die Zugangsdaten zur Krankenversicherung nutzen, um sich medizinische Dienstleistungen oder Medikamente zu erschleichen, vgl. zum sog. *Medical Identity Theft*, https://www.worldprivacyforum.org/2006/05/report-medical-identity-theft-the-information-crime-that-can-kill-you.

58 Siehe ausführlich zu „Neuronal Scanning“-Angriffen: Bernal et al. [93, S. 5 f., 14 ff.]. Das Scannen der Schnittstelle kann diese belasten und z. B. den Stimulationsprozess beeinträchtigen.

59 Z. B. sind ggf. Standardpasswörter bekannt oder ein Angreifer probiert Listen bekannter Passwörter durch [51, S. 109 f.].

60 Zur Verfügbarkeit im Datenschutzrecht siehe Mantz [94, Rn. 16].

61 Z. B. können Störsignale verhindern, dass die Schnittstelle echte neuronale Signale erkennt und verarbeitet (*Neuronal Jamming*) [95, S. 3 ff.].

62 Zur datenschutzrechtlichen Einordnung Mantz [94, Rn. 17] und Martini [77, Rn. 39].

63 Zur technischen Umsetzung eines *Neuronal Scannning*, der mittels Portscans die Verfügbarkeit und Belastbarkeit beeinträchtigen kann [93, S. 5 f., 14 ff.]. Zusätzlich kann eine hohe Auslastung dazu beitragen, dass sich die Batterie, die das Gerät betreibt, sehr viel schneller entlädt [39, S. 264; 92, S. 5].

64 Der Begriff der Integrität findet sich auch im Datenschutzrecht [77, Rn. 36; 94, Rn. 15; 96, Rn. 31]. Ein Überblick zu den zahlreichen Möglichkeiten, die Integrität von Gehirn-Computer-Schnittstellen anzugreifen, findet sich bei Bernal, Celdrán und Pérez [97, S. 3 ff.].

65 Daten lassen sich bereits bei der Aufnahme manipulieren, bspw. indem Eingriffe von außen den für bestimmte Reaktionen relevanten Stimulus beeinflussen. Zudem ist ein Eingriff in die Verarbeitung neuronaler Signale durch Manipulationen beim Messen der Gehirnaktivitäten oder in die Klassifizierung der Gehirnaktivität denkbar [74, S. 122 ff.].

66 Insbesondere können *Neuronal-Flooding*-Angriffe Neuronen überstimulieren [93, S. 5, 9 ff., 17 f.]. Die Möglichkeit, dem Nutzer Schmerzen oder einen erheblichen körperlichen Schaden zuzufügen, lässt sich auch zur Erpressung einsetzen [92, S. 3].

67 Neben klassischen Cyberattacken könnten Angreifer elektromagnetische Störungen nutzen, um z. B. Sensoren anzugreifen und deren Messungen zu verfälschen [92, S. 6].

68 D. h. via elektromagnetischer Wellen. Medizinprodukte nutzen ein Frequenzband von 401 bis 406 MHz (nach der *Medical-Implant-Communication-Service*-Spezifikation bzw. nach dem europäischen Standard ETSI EN 301 839-1). Innerhalb dieses Frequenzbandes „suchen“ sich die Geräte einen freien Kanal, auf dem sie dann Nachrichten bzw. Befehle zwischen dem steuernden Gerät und dem Implantat austauschen [99, S. 2 f.].

69 Microsoft unterstützt Windows XP bspw. nicht mehr. Das macht das System zum leichten Angriffsziel [102]. Siehe die Liste der (bekannten) Schwachstellen unter https://www.cvedetails.com/vulnerability-list/vendor_id-26/product_id-739/Microsoft-Windows-Xp.html. Der Wanna-Cry-Cyberangriff im Jahr 2017 veranlasste Microsoft jedoch dazu, selbst für Windows XP ein Sicherheitsupdate bereitzustellen [103].

70 Zu Clouddiensten und Datenpools in diesem Kontext vgl. Hornung und Sixt [24, S. 830 f.].

71 Siehe bereits unter 1.1.2.

72 Siehe Fn. 35.

73 Der Zugriff auf verschlüsselte Kommunikation bleibt weiterhin möglich. Der Angreifer hört jedoch nur ein Wirrwarr mit – es sei denn, er kann die Daten entschlüsseln. Hashwerte (d. h. kryptografische Prüfsummen der verschlüsselten Nachricht) des Verschlüsselungsalgorithmus „mD5“ (*Message Digest 5*) lassen sich mittlerweile in Datenbanken abgleichen und entschlüsseln, siehe https://md5decrypt.net/en.

74 Bspw. möchte Neuralink es ermöglichen, das Neuroimplantat mit dem Smartphone zu verbinden [106].

75 „[T]ablets and smartphones constitute a high potential entry port for malware and cyberattacks because of their clueless and careless use: the opening of e‑mail attachments of unknown senders, careless surfing in open WLAN networks and missing security updates represent a small section of the various strains …“ [100, S. 14]. Auch Apps weisen häufig Sicherheitslücken auf [107].

76 Vgl. auch *FDA Safety Communication*: Cybersecurity Vulnerabilities Affecting Medtronic Implantable Cardiac Devices, Programmers, and Home Monitors (21.03.2019), https://www.fda.gov/medical-devices/safety-communications/cybersecurity-vulnerabilities-affecting-medtronic-implantable-cardiac-devices-programmers-and-home.

77 So hat bspw. die *Koordinierungsgruppe Medizinprodukte* Leitlinien aufgestellt [71]. Die US-amerikanische Food and Drug Administration (FDA) hat ebenfalls Leitlinien zur Cybersicherheit nach Inverkehrbringen des Produkts veröffentlicht: *FDA Guidance Document*, Postmarket Management of Cybersecurity in Medical Devices (Dezember 2016), https://www.fda.gov/regulatory-information/search-fda-guidance-documents/postmarket-management-cybersecurity-medical-devices.

78 Das Strafrecht sanktioniert bereits Cyberangriffe auf vielfältige Weise. Zum einen sind Cyberangriffe auf die Vertraulichkeit, Integrität und Belastbarkeit der Daten (§§ 202a, 202b, 202c, 202d StGB) und der Gehirn-Computer-Schnittstelle als solche (§§ 303a, 303b StGB) strafbewehrt. Zum anderen sind viele der Schädigungen eines Opfers, die ein Cyberangriff verursacht, strafbar, z. B. die Tötung (§§ 211, 212, 222 StGB) oder Körperverletzung (§§ 223 f., 226, 227, 229 StGB). Auch wenn ein Täter die Gehirn-Computer-Schnittstelle so manipuliert, dass sie das Opfer zu Handlungen verleitet (§ 240 StGB) oder er das Opfer hierdurch bedroht oder erpresst (§§ 241, 239 bzw. 253 StGB), greift das Sanktionsinstrumentarium des Strafrechts [110, S. 254 ff.]. Siehe auch 2.1.2 sowie Fn. 99 und 105 f.

79 Wie effektiv Abschreckung bei der Verbrechensprävention ist, ist ohnedies umstritten [112, Rn. 28].

80 Bspw. werden Angriffe der Cyber-Spionage-Gruppe HAFNIUM der Volksrepublik China zugerechnet [113].

81 Zur neuroethischen Einordnung von Privatheit und „*Neurosecurity*“: [35, S. 4; 114, S. 67; 115, S. 35; 116, S. 31 f.].

82 Siehe zu „*Mental Privacy*“ im internationalen bzw. europarechtlichen Grund- und Menschenrechtsgefüge Ienca und Andorno [83, S. 12, 15 ff.] sowie Kemper [117].

83 BVerfG, Urteil v. 15.12.1983 – 1 BvR 209, BVerfGE 65, 1 (53, Rn. 177). Vgl. auch Di Fabio [118, Rn. 173].

84 BVerfG, Urteil v. 20.04.2016 – 1 BvR 966, 1140/09, BVerfGE 141, 220 (304 Rn. 210); BVerfG, Urteil v. 10.10.2007 – 1 BvR 370, 595/07, BVerfGE 120, 274 (314 f.). Vgl. auch Kunig und Kämmerer [119, Rn. 81] sowie Stinner [120, S. 97 f.]. Der Integritätsschutz ist dabei funktional auf die Vertraulichkeit bezogen und unterscheidet sich von dem sicherheitsrechtlichen Topos der „Integrität“ [121, Rn. 28]. Zur „Integrität“ im Kontext der Datensicherheit, siehe bspw. Martini [77, Rn. 36].

85 BVerfG, Urteil v. 09.10.2002 – 1 BvR 1611/96 u. a., BVerfGE 106, 28 (35 f.); Urteil v. 02.03.2006 – 2 BvR 2099/04, BVerfGE 115, 166 (182 f.); Urteil v. 10.10.2007 – 1 BvR 370, 595/07, BVerfGE 120, 274 (306 ff.); Urteil v. 24.01.2021 – 1 BvR 1299/05, BVerfGE 130, 151 (179 f.). Siehe auch Durner [122, Rn. 106]. Abzugrenzen ist das IT-Grundrecht zudem von dem Recht auf Unverletzlichkeit der Wohnung (Art. 13 Abs. 1 GG), das nur einen raumbezogenen Schutz eröffnet, BVerfG, Urteil v. 10.10.2007 – 1 BvR 370, 595/07, BVerfGE 120, 274 (310 f., v. a. Rn. 194).

86 Der Schutz des Art. 10 setzt aber einen Kommunikationsvorgang *zwischen verschiedenen Personen* voraus. Eine reine Maschinenkommunikation, die sich nicht zwischen zwei Menschen vollzieht, unterfällt nicht dem Schutzbereich des Art. 10 GG [123, Rn. 74 f.]. Die Datenübertragung zwischen einer Gehirn-Computer-Schnittstelle und bspw. einer Prothese ist folglich kein Kommunikationsvorgang.

87 Vgl. BVerfG, 02.03.2006 – 1 BvR 2099/04, BVerfGE 115, 166 (182). Zur Abgrenzung zwischen Art. 10 GG und dem IT-Grundrecht vgl. etwa Martini und Fröhlingsdorf [124, S. 3 ff.].

88 Eine Körperverletzung i. S. d. § 223 Abs. 1 StGB fügt der Täter dem Opfer zu, wenn er ihm Schmerzen verursacht oder dessen Körperfunktionen herabsetzt [127, Rn. 10, 13].

89 Auch das Zivilrecht erachtet Implantate als Teil des menschlichen Körpers (und damit nicht als Sache) [129, Rn. 28].

90 Zum Schutz „geistiger Integrität“: Herdegen [130, Rn. 95]. Die Möglichkeit, das Gehirn bzw. seine Aktivitäten zu manipulieren, stellt das Konzept des freien Willens [52, S. 3] und die Verantwortlichkeit der Nutzer für ihr Verhalten auf die Probe [4, S. 197]; zu möglichen Schwierigkeiten im Zusammenhang mit Kausalität und Beweisbarkeit: Gasson und Koops [39, S. 265].

91 Die genaue Qualifikation mentaler Vorgänge und Zustände ist in der Philosophie des Geistes ein vieldiskutiertes, wissenschaftlich bislang noch nicht eindeutig erklärbares Thema; siehe zum Überblick z. B. Flanagan [132, S. 603 ff.].

92 In diese Richtung argumentiert Lindner [126, S. 466]. Zu „Cognitive Liberty“ siehe Farahany [133, S. 98 ff.].

93 So verortet Lindner [126, S. 466 f.] den Schutz der Autonomie bei der Menschenwürde: Diese schütze „den Einzelnen generell vor einer ohne oder gegen seinen Willen erfolgenden Veränderung seines mentalen So-Seins und konkret davor, sich einer […] Fremdmanipulation unterziehen oder aussetzen zu müssen.“ Die Verankerung in Art. 1 Abs. 1 GG habe „zur Folge, dass ein fremdbestimmter Eingriff des Staates keiner Rechtfertigung zugänglich ist und ein fremdbestimmter Eingriff seitens Dritter die staatliche Schutzpflicht auslöst, diesen zu verhindern, notfalls durch das Strafrecht“.

94 Um ein klares Schutzgut zu etablieren, fordern *Andorno* und *Ienca* (im Kontext internationaler Menschenrechte), ein eigenständiges Recht auf mentale Integrität zu verbriefen [83, S. 17 ff.].

95 Fragen der Autonomie diskutiert die neuroethische Literatur rege [74, S. 127 f.; 78, S. 225 ff.; 114, S. 6; 135, S. 622 f.;].

96 Das BVerfG hebt die Bedeutung des dynamischen Grundrechtsschutzes gerade in Bezug auf moderne bzw. technische Entwicklungen hervor; BVerfG, Beschluss v. 03.06.1980 – 1 BvR 185/77, BVerfGE 54, 148 (153). Den Stellenwert insbesondere der Privatsphäre und der Privatheit für Autonomie entwickelt Matwyshyn [101, S. 159 ff.] aus dem Konzept der *Heautonomie* von *Immanuel Kant* als die (Willensbildungs‑)Phase, die der Autonomie vorgelagert ist. Zum Verhältnis von Intimität und Sozialität siehe Di Fabio [118, Rn. 129].

97 Die Erfahrungen einzelner Patienten bei rehabilitativen Gehirn-Computer-Schnittstellen können dabei sehr unterschiedlich ausfallen: Manche fühlen sich (wieder) wie sie selbst, andere empfinden einen Kontrollverlust und die Gehirn-Computer-Schnittstelle letztlich als störenden Fremdkörper [136].

98 Obgleich Art. 2 Abs. 2 S. 2 GG die Bewegungsfreiheit schützt, ist sein Schutzbereich eng und das Grundrecht v. a. als Abwehrrecht gegen den Staat konzipiert [137, Rn. 20, 22, 24 f.]. Allerdings kann eine Freiheitsberaubung i. S. d. § 239 Abs. 1 StGB vorliegen, wenn man z. B. Menschen mit Behinderung Hilfsmittel entzieht. Denn der Angriff höbe die Fortbewegungsfreiheit des Betroffenen auf und hinderte ihn daran, seinen Aufenthaltsort zu verlassen [138, Rn. 26 f.]. § 239 StGB soll gerade die persönliche Fortbewegungsfreiheit bzw. das Selbstbestimmungsrecht der Person über ihren Aufenthaltsort schützen [138, Rn. 1].

99 Wenn Nutzer ihre Schnittstellen als Teil des eigenen Körpers verstehen, stellt sich die Frage, ob diese Erweiterungen Teil des Körpers (Art. 2 Abs. 2 S. 1 GG) oder der Identität sind (Art. 2 Abs. 1 i. V. m. Art. 1 Abs. 1 GG).

100 Vgl. auch Kersten [140, S. 4]. Das Recht am Eigentum schützt auch das Recht, von dem Gegenstand Gebrauch zu machen [139, Rn. 146]. Kersten [140, S. 4] wirft die Frage auf, ob das Recht auf körperliche Unversehrtheit bspw. auch die symbiotische Verbindung eines Locked-in-Patienten mit einem EEG-Gerät schützt. Gleichzeitig kann das Eigentumsrecht des Herstellers verletzt sein, wenn der Angreifer z. B. durch *Reverse Engineering* Sicherheitslücken findet und dabei das Urheberrecht des Herstellers am Computerprogramm verletzt (§ 69c, 69d Abs. 3 UrhG) [141, Rn. 21].

101 Zu DoS-Angriffen, siehe bereits 1.2.2.a)bb).

102 BVerfG, Urteil v. 25.02.1975 – 1 BvF 1/74 u. a., BVerfGE 39, 1 (42); siehe auch Alexy [143, S. 410 ff.].

103 Das BVerfG bestätigte dies im Beschluss v. 08.06.2021 – 1 BvR 2771/18, Rn. 33, NJW 2021, 3033 (3035).

104 Welches Rechtsgut der Straftatbestand aus § 202a StGB schützt, ist indes umstritten [145, Rn. 1]. In Rede steht insbesondere das formelle Datengeheimnis, das in Bezug auf § 202b StGB eine besondere Ausprägung des Art. 10 GG darstellt [146, Rn. 2]. Hinzu treten auch die Strafbarkeit der Datenhehlerei (§ 202d StGB) sowie der Vorbereitung des Ausspähens und Abfangens von Daten (§ 202c StGB).

105 Schutzgut der §§ 303a, 303b StGB ist die Integrität der Computerdaten bzw. des Computersystems [147, Rn. 1; 148, Rn. 1].

106 Zum strafrechtlichen Schutz von Herrschaftspositionen über Daten siehe auch Martini et al. [149, S. 14 ff.].

107 Ziel der Cybersicherheit ist es, Netz- und Informationssysteme sowie deren Nutzer oder potenziell betroffene Personen zu schützen, vgl. auch Art. 2 Abs. 2 der Verordnung (EU) 2019/881 des Europäischen Parlaments und des Rates vom 17. April 2019 über die ENISA (Agentur der Europäischen Union für Cybersicherheit) und über die Zertifizierung der Cybersicherheit von Informations- und Kommunikationstechnik.

108 Siehe hierzu 2.2. Neben dem Haftungsrecht (insbesondere § 823 Abs. 1 BGB) ist vor allem der schuldrechtliche Anspruch auf Softwareaktualisierung von Bedeutung, den der Gesetzgeber zur Umsetzung der neuen Warenverkauf-Richtlinie (EU) 2019/771 eingeführt hat (siehe hierzu 2.2.3. sowie Fn. 215 ff.). Das Produkthaftungsregime dagegen greift nur, wenn Cyberangriffe Personen- oder Sachschäden verursachen (§ 1 Abs. 1 S. 1 ProdHaftG). Die Ersatzpflicht des Herstellers ist auch in vielen Fällen deshalb ausgeschlossen, weil die Sicherheitslücke beim Inverkehrbringen nicht vorhersehbar gewesen und ggf. erst im Nachhinein entstanden ist (§ 1 Abs. 2 Nr. 2, Nr. 5 ProdHaftG).

109 Der Gesetzgeber darf seiner Schutzpflicht nur gegenüber der Allgemeinheit nachkommen, dem sich selbst gefährdenden Einzelnen aber nicht ohne Weiteres Schutz aufdrängen (vgl. auch BVerfG, Urteil vom 30.07.2008 – 1 BvR 3262/07 u. a., Rn. 126, NJW 2008, 2409 [2414]). Wenn dieser nicht nur sich selbst schadet, kann der Gesetzgeber ihm aber Pflichten auferlegen. „Wenn die Folgen eines im öffentlichen Straßenverkehr eingegangenen, berechenbaren und hohen Risikos die Allgemeinheit schwer belasten, ist es für den einzelnen zumutbar, dieses Risiko durch einfache, leicht zu ertragende Maßnahmen zu senken“, BVerfG, Beschluss vom 26.01.1982 – 1 BvR 1295/80 u. a., NJW 1982, 1276. Siehe hierzu im Kontext von Enhancement auch Hornung und Sixt [150, S. 130].

110 Die Selbstgefährdung kann andere beeinträchtigen, wenn z. B. Rettungsdienste oder die Sozialversicherung helfend einspringen [125, Rn. 85].

111 Siehe zum Selbstbestimmungsrecht über die körperliche Integrität auch Lang [151, Rn. 63].

112 *Lindner* nimmt an, dass körperliche Eingriffe mit dem Ziel des Neuroenhancement in den Schutzbereich der allgemeinen Handlungsfreiheit fallen. Neuroenhancement zu betreiben, sei demgegenüber dem Grundrecht auf neuronale Selbstbestimmung zuzuordnen, das er aus Art. 2 Abs. 1 i. V. m. Art. 1 Abs. 1 GG entwickelt [126, S. 466 f.]. Zudem könnte Neuroenhancement in der „Biohacker“-Szene [10, S. 2 f.] unter die Forschungsfreiheit fallen [126, S. 467 f.].

113 BVerfG, Beschluss v. 09.03.1994 – 2 BvL 43/92 u. a., BVerfGE 90, 145 (171 f.). Vgl. auch Starck [152, Rn. 126].

114 Erfolgt ein *Neuroenhancement*-Eingriff ohne medizinische Indikation, stellen sich langfristig die gleichen Fragen, die schon die ästhetisch-plastische Chirurgie aufgeworfen hat [153]. So könnte es neben der medizinischen und ästhetischen Indikation bald eine „Enhancement-Indikation“ geben.

115 Gewährleisten Verbrauchergeräte keine ausreichende IT-Sicherheit, stellt sich zudem bereits die Frage, ob sich die Nutzer überhaupt eigenverantwortlich selbst gefährden – oder nicht vielmehr einer Gefährdung ausgesetzt sind. Entsprechend der Abgrenzung zwischen Fremd- und Selbstgefährdung im Strafrecht – ist insoweit auf die *Gefährdungsherrschaft* abzustellen. Kraft überlegener Sachkenntnis hat diese grundsätzlich der Hersteller inne [154, Rn. 27, 29].

116 Im deliktischen (Produkt‑) Haftungsrecht darf der Verbraucher jedenfalls eine „Basissicherheit“ erwarten, die den bestimmungsgemäßen Einsatz des Produkts überhaupt erst erlaubt [155, Rn. 687 f., 692]. Um der „berechtigten Erwartung“ klare Konturen zu verleihen, ist der „Stand der Technik“ ein zentraler Konkretisierungsmaßstab [156, S. 3347].

117 Z. B. bietet *Open BCI* solche Geräte an, vgl. https://shop.openbci.com/collections/frontpage; siehe auch die (unterdessen veraltete) Seite von OpenEEG, http://openeeg.sourceforge.net/doc/. Der Einpflanzung von DIY-Neuroimplantaten lässt sich angesichts der erforderlichen Schädelöffnung mit den strafrechtlichen Normen, insbesondere § 228 StGB, begegnen [150, S. 132].

118 Dies folgt aus dem Grundrecht auf körperliche Unversehrtheit (Art. 2 Abs. 2 S. 1 GG) i. V. m. dem Sozialstaatsprinzip: Existenzrisiken und die medizinische Notfallversorgung hat die Solidargemeinschaft zu tragen – obgleich aus den Grundrechten sonst keine konkreten Leistungsansprüche des Einzelnen erwachsen [125, Rn. 94].

119 Allerdings unterfallen nicht jegliche Daten, aus denen sich möglicherweise Informationen über den Gesundheitszustand einer Person ziehen lassen, dem grundsätzlichen Verarbeitungsverbot aus Art. 9 Abs. 1 DSGVO [160, Rn. 9; 161, S. 259; 162]. Vielmehr muss sich das Datum auf die Gesundheit beziehen: Aus ihm muss unmittelbar eine Information über den Gesundheitszustand hervorgehen. Fungiert die Gehirn-Computer-Schnittstelle bspw. nur als Steuerungsinstrument (so ermöglicht es bspw. die App „BlinkReader“ Gelähmten, in E‑Books durch Blinzeln zu blättern, https://store.neurosky.com/products/blinkreader), ohne die verarbeiteten Gehirndaten weiter gehend zu analysieren, fallen diese aus dem Anwendungsbereich des Art. 9 Abs. 1 DSGVO heraus.

120 Siehe z. B. die App „Calme“, https://store.neurosky.com/products/calme.

121 Der Begriff der Gesundheitsdaten ist grundsätzlich weit auszulegen (Art. 4 Nr. 15 sowie Erw. gr. 35 der DSGVO); siehe auch EuGH, Urteil v. 06.11.2003 – C‑101/01, EuZW 2004, 245 (249, Rn. 50). Wahrscheinlichkeitsaussagen oder Vermutungen fallen ebenfalls hierunter, selbst bei fraglicher Verlässlichkeit der Daten [163, S. 4].

122 Siehe bereits 1.1.2., aber auch Ernst [164, Rn. 99].

123 Datensicherheit im Datenschutzrecht [77, Rn. 25 ff.] unterscheidet sich von der IT-Sicherheit. Diese nimmt weniger die von der Datenverarbeitung Betroffenen, sondern die IT-Systeme selbst in den Blick [165, S. 463 f.; 166, S. 160 f.].

124 Zum Datenschutzmanagement allgemein siehe bspw. Martini [168, Rn. 40] sowie das „Standard-Datenschutzmodell“ des Unabhängigen Landeszentrum für Datenschutz, https://www.datenschutzzentrum.de/sdm/.

125 Das Niveau der technischen und organisatorischen Maßnahmen muss „dem Risiko angemessen“ sein: Zu berücksichtigen sind zum einen die mit der Verarbeitung verbundenen Risiken, insbesondere die Eintrittswahrscheinlichkeit und Schwere; zum anderen fließen die Besonderheiten des Verarbeitungsprozesses, aber auch praktische Erwägungen zum Stand der Technik und den Implementierungskosten der Maßnahmen in die Wertung ein [77, Rn. 46 ff.].

126 Entscheidend ist nicht nur, ob der Verantwortliche die Daten als Gehirndaten verarbeitet, sondern auch das Missbrauchspotenzial. Wenn zu erwarten ist, dass unbefugte Dritte diese Daten ausspähen, um Informationen über den Gesundheitszustand des Betroffenen abzuleiten (selbst, wenn die legitime Datenverarbeitung keinen Gesundheitsbezug aufweist), ist das Schutz*niveau* der Gehirndaten an ihre Schutz*bedürftigkeit* als potenzielle Gesundheitsdaten anzupassen. Dass diese Daten verändert, gelöscht oder offengelegt werden, mag derzeit unwahrscheinlich erscheinen; jedoch sind die Schadensszenarien von Rufschädigung und gesellschaftlichen Nachteilen bis hin zu finanziellen Verlusten sehr sensibel und vielfältig [77, Rn. 52 ff.].

127 https://www.iso.org/isoiec-27001-information-security.html. Experten wirken bei der Entwicklung von Standards mit und nehmen maßgeblich Einfluss auf diese. Gerade im Bereich der Cybersicherheitsstandards verlagern sich Normsetzungskompetenzen auf Private [169].

128 Das IT-Grundschutz-Kompendium stellt umfassend Gefahrenszenarien sowie Prozess- und System-Bausteine für ein (Informations‑) Sicherheitsmanagement zusammen [154 ISMS.1; 170].

129 Vgl. die Anforderungen des IT-Grundschutz-Kompendiums [172], z. B. APP.6.A6 und SYS.4.4.A11 (für IoT-Geräte). Implantierte Geräte, die keine Ende-zu-Ende-Verschlüsselung zulassen, sind grundsätzlich auszutauschen, soweit sie sich nicht nachrüsten lassen [99]. Gerade eine asymmetrische Verschlüsselung lässt sich schwerer nachrüsten, da sie rechenintensiver ist. Bei symmetrischer Verschlüsselung müssen die Schlüssel vorher unter den Beteiligten verteilt werden [92, S. 8].

130 Siehe auch die allgemeinen Anforderungen des BSI IT-Grundschutz-Kompendiums [172]: ORP.4.A7, APP.6.A6 und SYS.1.1.A2 (für allgemeine Software bzw. Server) sowie SYS.4.4.A2, SYS.4.4.A15 und APP.1.4.A7. Bei erhöhtem Schutzbedarf sollte die App zusätzliche Authentisierungsmerkmale unterstützen (APP.1.4.A14).

131 Zur Vergabe *sicherer* Passwörter, siehe bspw. https://www.verbraucherzentrale.de/wissen/digitale-welt/datenschutz/sichere-passwoerter-so-gehts-11672.

132 Siehe https://www.bsi.bund.de/DE/Themen/Verbraucherinnen-und-Verbraucher/Informationen-und-Empfehlungen/Cyber-Sicherheitsempfehlungen/Accountschutz/Zwei-Faktor-Authentisierung/zwei-faktor-authentisierung_node.html.

133 Siehe auch SYS.1.1.A27 (zur hostbasierten Angriffserkennung) sowie die Anforderungen für IoT-Geräte SYS.4.4.A16 zu Beseitigung von Schadprogrammen [172]. Dies bringt wiederum datenschutzrechtliche Probleme mit sich, wenn das Detektionssystem Aktivitäten verschiedener Personen speichert und analysiert [165, S. 466 ff.].

134 Die Detektionssoftware erkennt Schadprogramme entweder anhand ihrer Signatur oder aufgrund von Anomalien [23, S. 138, 152 f.].

135 Siehe z. B. die „Security Event Manager“-Software von SolarWinds, https://www.solarwinds.com/security-event-manager/use-cases/ddos-attack (aber auch CVE-2021-35211). Diese Funktionen verursachen datenschutzrechtliche Konflikte [165, S. 464 ff.]. Vgl. auch SYS.4.4.A17 zur Überwachung des Netzverkehrs und SYS.4.4.A18 zur Protokollierung sicherheitsrelevanter Ereignisse bei IoT-Geräten bzw. SYS.4.3.A3 bei sonstigen Systemen [172].

136 Mit Methoden des maschinellen Lernens kann *Intrusion Detection Software* den Datenverkehr auswerten, um Angriffsmuster zu erkennen [173].

137 Zum sog. *BCI Anonymizer* (Patent US20140228701A1, https://patents.google.com/patent/US20140228701A1/en), siehe Bonaci, Calo und Chizeck [14, S. 37 f.]. Umsetzbar wäre dieses Modell auf Software- oder Hardwareebene.

138 Der Begriff „Hacker“ ist weit. Er beschreibt (bei offenem Verständnis) Menschen, die komplexe technische Probleme lösen und hierbei die Grenzen des Möglichen austesten, vgl. https://koeln.ccc.de/ablage/artikel/hacker-howto-esr.xml. Die Hacker-Kultur entstand in den 1960er-Jahren u. a. am *Massachusetts Institute of Technology*, wo Studenten z. B. Streiche spielten oder sich Zugriff auf gesperrte Bereiche verschafften [179, S. 14 f.; 180, S. 11]; siehe auch http://hacks.mit.edu/misc/faq.html.

139 „While it is impossible to envision all of the ways that hackers will find to hack into and take advantage of [implanted medical devices], one thing is certain: if there is money to be made in hacking, the law of supply and demand will ensure there will be economic incentive for the hacks to continue“ [76, S. 662].

140 *Defense in Depth* erfordert viele Schichten von Sicherheitsmaßnahmen, u. a. menschliche und organisatorische Faktoren [51, S. 7, 71 f.]. Die Koordinierungsgruppe Medizinprodukte schlägt ebenfalls eine *Defense-in-Depth*-Strategie vor [71, S. 15 f.].

141 Das IT-Grundschutz-Kompendium [172] enthält zahlreiche Anforderungen an Desktop-Systeme (SYS.2), Smartphones (SYS.3.2), usw.

142 Im „Internet of Medical Things“ sind dynamischen Sicherheitsupdates sinnvoll [182, S. 40 f.]. Allerdings bergen Sicherheitsupdates die Gefahr, neue Probleme und Schwachstellen zu verursachen [183, S. 5, 44].

143 Ein Penetrationstest ist ein simulierter Angriff, der ein Programm oder System in einer Testumgebung auf Schwachstellen prüft und untersucht, wie sich die Schwachstelle wirksam ausnutzen lässt, indem der Täter das Verhalten typischer Angreifer nachahmt [51, S. 46].

144 Red Teams versuchen, das System anzugreifen und strategisch Schwachstellen auszunutzen [184].

145 Siehe im IT-Grundschutz-Kompendium [172] OPS.1.1.6.A14 und APP.1.4.A15 sowie Martini [77, Rn. 44].

146 Unter Umständen entfällt die Pflicht, Betroffene zu benachrichtigen, wenn die Personenzahl so hoch ist, dass dies einen unverhältnismäßigen Aufwand mit sich brächte (Art. 34 Abs. 3 lit. c DSGVO). Der Verantwortliche muss dann eine öffentliche Bekanntmachung oder eine „ähnliche“ Maßnahme wählen [77, Rn. 40 ff.].

147 Das IT-Grundschutz-Kompendium [172] enthält konkretisierende Empfehlungen zur Klärung der Prozesse und Verantwortlichkeiten, siehe u. a. DER.2.1.A2, DER.2.1.A3, DER.2.1.A7 und DER2.1.A8, sowie Anforderungen, um weitreichende Sicherheitsvorfälle zu bereinigen (DER.2.3): So müssen die Systemverantwortlichen die Zugangsdaten und kryptografische Schlüssel sperren bzw. ändern (DER.2.3.A4) und den initialen Einbruchsweg schließen (DER.2.3.A5), sodass sie die Systeme anschließend geordnet wieder in den Produktivbetrieb zurückführen können (DER.2.3.A6).

148 Erw. gr. 78 S. 4 DSGVO verdeutlicht, dass der Unionsgesetzgeber Hersteller *ermutigen* will, „das Recht auf Datenschutz bei der Entwicklung und Gestaltung der Produkte, Dienste und Anwendungen zu berücksichtigen und unter gebührender Berücksichtigung des Stands der Technik sicherzustellen, dass die Verantwortlichen und die Verarbeiter in der Lage sind, ihren Datenschutzpflichten nachzukommen“.

149 Verordnung (EU) 2017/745 des Europäischen Parlaments und des Rates vom 5. April 2017 über Medizinprodukte, zur Änderung der Richtlinie 2001/83/EG, der Verordnung (EG) Nr. 178/2002 und der Verordnung (EG) Nr. 1223/2009 und zur Aufhebung der Richtlinien 90/385/EWG und 93/42/EWG des Rates [187]. Das nationale Medizinprodukte-Durchführungsgesetz (MPDG) enthält ergänzende Bestimmungen.

150 Die Verordnung (EU) 2020/561 verschob den Geltungsbeginn wegen der COVID-19-Pandemie um ein Jahr [188; 189, S. 131 f.].

151 Ein „implantierbares Produkt muss dazu bestimmt sein, ganz in den menschlichen Körper eingeführt zu werden und nach dem Eingriff dort zu verbleiben“ (Art. 2 Nr. 5 S. 1 Spiegelstrich 1 MPVO).

152 Neuroimplantate gehören der Risikoklasse III an, siehe Regel 8, Anhang VIII Nr. 5.4 Spiegelstrich 2 MPVO. Es handelt sich um *chirurgisch-invasive Produkte* (d. h. invasive Produkte, die mittels eines chirurgischen Eingriffs in den Körper eindringen, Anhang VIII Nr. 2.2. a Alt. 1 MPVO), die dazu bestimmt, *in direktem Kontakt mit dem zentralen Nervensystem* (d. h. mit dem Gehirn, der Hirnhaut oder dem Rückenmark, Anhang VIII Nr. 2.7. MPVO) Verwendung zu finden. Ausnahmen gibt es bei Implantaten, die lediglich dazu dienen, Anomalien aufzufinden, z. B. als Ursprung für Epilepsieanfälle, und die nur für bis zu 30 Tage eingepflanzt werden (Anhang VIII Nr. 1.2 MPVO). Nach der Regel 7 (Anhang VIII Nr. 5.3. MPVO) gehören sie zur Klasse IIa.

153 Als aktive, nicht invasive Medizinprodukte sind sie in Klasse IIa einzuordnen. Transkraniell stimulierende Geräte gehören der Klasse IIb an, vgl. Regel 9 bzw. 10, Anhang VIII Nr. 6.1. und 6.2. MPVO.

154 Vgl. Anhang VIII Nr. 3.3 MPVO.

155 Regel 11, Anhang VIII Nr. 6.3 MPVO; siehe auch die Definition des International Medical Device Regulators Forum [190]. Zu den medizinprodukterechtlichen Besonderheiten, die sich aus der Lernfähigkeit der Software ergeben, siehe Gassner [191, S. 44 ff.].

156 „Zweckbestimmung“ bezeichnet die Verwendung, für die ein Produkt – entsprechend den Angaben des Herstellers auf der Kennzeichnung, in der Gebrauchsanweisung oder dem Werbe- oder Verkaufsmaterial bzw. den Werbe- oder Verkaufsangaben und seinen Angaben bei der klinischen Bewertung – bestimmt ist (Art. 2 Nr. 12 MPVO) [193, Rn. 1]. Der BGH, Urteil v. 18.04.2013 – I ZR43/09, hat daher ein EEG-System, dessen Hersteller die Verwendung für einen medizinischen Zweck unmissverständlich ausgeschlossen hatte, nicht als „Medizinprodukt“ eingestuft (a. a. O., juris, Rn. 8). Das Produkt sei überdies ausschließlich darauf gerichtet, physiologische Vorgänge beim Menschen außerhalb einer krankheits- oder gesundheitsbezogenen Verwendung zu beobachten (juris, Rn. 14). Siehe auch das der Vorlagefrage des BGH vorhergehende Urteil des EuGH: EuGH, Urteil v. 22.11.2012 – C‑219/11.

157 Vgl. z. B. die Geräte von OpenBCI (https://shop.openbci.com/collections/frontpage/products/openbci-eeg-electrocap) oder Emotiv (https://www.emotiv.com/insight/).

158 Wenn künftig invasivere, nichtmedizinische Produkte auf den Markt strömen, ist aber „zu analysieren, ob es dauerhaft angemessen ist, reine Enhancement-Systeme allein dem allgemeinen Produkthaftungs- und Datenschutzrecht zu überlassen“ [24, S. 837].

159 Siehe bereits Fn. 8.

160 In Bezug auf „Wunschmedizin“ ohne medizinische Indikation, vgl. Damm [153, S. 645 f.].

161 Die Vorgängerregelungen der Medizinprodukte-Richtlinie und des MPG enthielten diese Ausnahme noch nicht [44, S. 73 ff.].

162 Anhang XVI Nr. 6 MPVO sollte für Produkte zur intrakraniellen Stimulierung erst recht gelten, da diese implantiert werden und somit invasiver sind. An die Fähigkeit des Implantats, sich in das Hirngewebe einzufügen, sowie an die langfristige Verträglichkeit des Materials sind ohnehin besondere Anforderungen zu stellen [1, S. 22; 2, S. 780; 195]. Die Europäische Kommission wusste um die Lücke, die bereits die Medizinprodukte-Richtlinie 93/42/EWG im Bereich der Enhancement-Produkte hinterlassen hatte. Dennoch entschied sie sich in der MPVO, auf den medizinischen Zweck abzustellen und dafür in Anhang XVI der MPVO-Produkte auszunehmen, auf die sich die MPVO erstrecken soll [196, S. 6], z. B. Produkte zur transkraniellen Stimulierung (vgl. die Kritik zur Medizinprodukte-Richtlinie von Maslen et al. [44, S. 80 ff.]). Es liegt daher nahe, Anhang XVI in Zukunft um Neuroimplantate zu erweitern.

163 Beispiele hierfür sind Prothesen, künstliche Gelenke oder kosmetische Implantate, welche Teile der Anatomie des Patienten ersetzen oder verändern. § 3 Nr. 1 lit. c MPG a. F. nannte Produkte, die dem Zweck dienen, den anatomischen Aufbau oder einen physiologischen Vorgang zu untersuchen, zu ersetzen oder zu verändern [193, Rn. 6; 194, Rn. 5].

164 Neuroplastische Veränderungen gehen insbesondere auf „Lerneffekte“ zurück: Häufig stattfindende neuronale Aktivitäten intensivieren die Verbindungen zwischen den hierfür relevanten Neuronen. Trainiert ein Nutzer seine Gehirn-Computer-Schnittstelle bspw., um bestimmte Bewegungen mit einer Prothese durchzuführen oder um einen Sprachcomputers zu bedienen, werden die beanspruchten Neuronengruppen sich so organisieren, dass die Aktivität künftig leichter und schneller durchgeführt werden kann. Zur Neuroplastizität vgl. allgemein Bear, Connors und Paradiso [32, S. 882 ff.].

165 Andere Mensch-Maschine-Schnittstellen, v. a. Eingabegeräte wie Maus und Tastatur, wären dann redundant bzw. stünden bei Störungen als eine zusätzliche Steuerungsmöglichkeit zur Verfügung.

166 Solchen Technologien werfen Fragen der Verteilungsgerechtigkeit und des Zugangs auf [114, S. 8; 197, S. 610].

167 Die Europäische Kommission könnte insbesondere auf der Grundlage des Art. 1 Abs. 5 MPVO einen delegierten Rechtsakt erlassen und Produkte zu Anhang XVI hinzufügen, die in Verkehr gebrachten Produkten mit medizinischer Zweckbestimmung ähnlich sind und die damit verbundenen Risiken rechtfertigen.

168 Vgl. § 21 Abs. 1 S. 1 AMG. Siehe auch Schmidt [198, S. 701].

169 Eine klinische Bewertung (Art. 2 Nr. 44 MPVO) ist jedenfalls bei Neuroimplantaten erforderlich, die der Risikoklasse III angehören [200, S. 186 f.].

170 Die Behörden kontrollieren „nicht die Produkte, sondern die Prüfer“, d. h. die Benannten Stellen (Art. 2 Nr. 42, Art. 35 ff. MPVO). Bei bestimmten implantierbaren Medizinprodukten findet zudem ein Konsultationsverfahren mit einem Expertengremium statt (Art. 54 MPVO). Wenn ein Produkt harmonisierten Normen entspricht, wird die Konformität eines Produkts mit der MPVO hingegen fingiert (Art. 8 Abs. 1 MPVO). Z. B. formuliert die Norm EN 60601-2-26:2003 Anforderungen an die Sicherheit von EEGs und EN 45502-2-3:2010 an Cochleaimplantate, vgl. ABl. EU 2020, Nr. L 090I, S. 1 ff., 25 ff. Das Konformitätsverfahren ist Teil des „New Legislative Framework“ [201, Rn. 82e]. Kritisch hierzu: Veale und Zuiderveen Borgesius [202, S. 104 ff.].

171 Vgl. Art. 52 Abs. 1, Abs. 2 MPVO i. V. m. Anhang IX.

172 Zum Verhältnis von „*Safety and Security*“ vgl. die Leitlinien der Koordinierungsgruppe Medizinprodukte [71, S. 9 f.]. Schwache (IT-)Sicherheit gefährdet die Sicherheit der Patienten wegen der Gefahr einer Cyberattacke; zu restriktive (IT-)Sicherheit kann die Patientensicherheit dadurch gefährden, dass medizinisches Personal im Notfall nicht mehr ohne Weiteres auf das Medizinprodukt zugreifen kann.

173 Die Hersteller legen zudem Mindestanforderungen zur „Hardware, Eigenschaften von IT-Netzen und IT-Sicherheitsmaßnahmen einschließlich des Schutzes vor unbefugtem Zugriff fest, die für den bestimmungsgemäßen Einsatz der Software erforderlich sind“ (Anhang I Nr. 17.4. MPVO). Außerdem müssen sie in der Gebrauchsanweisung Angaben zu Mindestanforderungen zur Hardware, Eigenschaften der eingesetzten IT-Netze und IT-Sicherheitsmaßnahmen einschließlich des Schutzes vor unbefugtem Zugriff, machen, die für den bestimmungsgemäßen Einsatz der Software erforderlich sind (Anhang I Nr. 23.4. MPVO).

174 Die Koordinierungsgruppe Medizinprodukte geht auf Art. 103 der Medizinprodukte-Verordnung (EU) 2017/745 zurück. Sie soll u. a. bei der Entwicklung von Normen, gemeinsamen Spezifikationen und Leitlinien für die klinische Prüfung bestimmter Produkte (Art. 105 lit. e) sowie für die Anwendung der grundlegenden Sicherheits- und Leistungsanforderungen (Art. 105 lit. c) mitwirken.

175 Vgl. die Leitlinien zur Cybersicherheit [71, S. 20] sowie Annex I [71, S. 35 ff.]. Siehe auch die Auslegungshilfe für die Anforderungen in Anhang I Nr. 17.4. und 23.4. MPVO [71, S. 20]. Zudem formuliert Annex II [71, S. 39 ff.] Anforderungen an Gebrauchsanweisungen [71, S. 24 ff.], Informationen an Gesundheitsdienstleister [71, S. 27 f.] sowie Beispiele.

176 Bislang sind nur wenige sekundäre Rechtsakte zur Anwendung der MPVO ergangen. Eine Ermächtigung findet sich u. a. in Art. 9 Abs. 1 MPVO (gemeinsame Spezifikationen für die grundlegenden Sicherheits- und Leistungsanforderungen) und Art. 91 MPVO (zur Umsetzung der Überwachung nach Inverkehrbringen), vgl. Hill [189, S. 130].

177 Z. B. Testen der Sicherheitsfunktionen, Fuzzy Testing, Sicherheitslücken-Scanning oder Penetrationstests [71, S. 22].

178 Die Verantwortung tragen die verschiedenen Akteure, d. h. Hersteller, Zulieferer, Gesundheitsdienstleister, Patienten, und ggf. Betreiber, gemeinsam [71, S. 12 ff.].

179 Weitere Maßnahmen enthält Annex I [71, S. 35 ff.], zur Umsetzung der NIS-Richtlinie (EU) 2016/1148 des Europäischen Parlaments und des Rates vom 6. Juli 2016 über Maßnahmen zur Gewährleistung eines hohen gemeinsamen Sicherheitsniveaus von Netz- und Informationssystemen in der Union.

180 Hersteller schienen lange auf das Prinzip „Security through Obscurity“ zu setzen und darauf zu vertrauen, dass potenzielle Angreifer das System und den Programmcode nicht kennen. Dabei erhöhen Passwörter die Sicherheit, ohne übermäßige Beschwernisse zu verursachen [76, S. 650].

181 Dies ist v. a. deshalb problematisch, weil es häufig unkompliziert möglich ist, die Serien- oder Modellnummer eines Geräts herauszufinden. Das passende Standardpasswort lässt sich dann mühelos im Internet finden [39, S. 264]. Das Problem ist im Kontext des Internets der Dinge gut dokumentiert; siehe z. B. die Angriffe der Gruppe Fancy Bear [205].

182 „*Globale*“ Gehirn-Computer-Schnittstellen könnten bspw. über eine Internetverbindung mit einer Cloud kommunizieren [45, S. 21 ff.]. Demgegenüber nutzen „*lokale*“ Gehirn-Computer-Schnittstellen Nahbereichskommunikation, um sich mit einem Gerät zu verbinden [45, S. 18 ff.].

183 Viele Schwachstellen sind unbekannt und noch nicht identifiziert, aber die Ausnutzung bekannter Schwachstellen kann als „vorhersehbar“ erachtet werden [71, S. 19].

184 Siehe dazu 1.2.2.

185 Hersteller sollen ein System zur Überwachung nach dem Inverkehrbringen (*Post-Market Surveillance System*) einrichten, das Daten über die Qualität, die Leistung und die Sicherheit eines Produkts während dessen gesamter Lebensdauer sammelt, aufzeichnet und analysiert. Diese Datenbasis erlaubt es, etwaige Präventiv- und Korrekturmaßnahmen zu ermitteln, durchzuführen und zu überwachen (vgl. Art. 83 Abs. 2 MPVO). Ein regelmäßig aktualisierter Sicherheitsbericht enthält Ergebnisse und Schlussfolgerungen sowie eine Begründung und Beschreibung etwaiger ergriffener Präventiv- und Korrekturmaßnahmen, Art. 86 Abs. 1 MPVO.

186 Gemäß Art. 89 Abs. 3 MPVO bewertet die Behörde die Risiken des gemeldeten Vorkommnisses, die Sicherheitskorrekturmaßnahmen im Feld sowie die Angemessenheit aller Korrekturmaßnahmen, die der Hersteller ergriffen hat. Zuständig ist das Bundesinstitut für Arzneimittel und Medizinprodukte (§ 85 Abs. 2 Nr. 12 MPDG).

187 Der Hersteller muss hierfür einen *Patch-Management*-Prozess einführen, durch den sich Aktualisierungen zügig und ohne Konflikte mit der Betriebsumgebung des Medizinprodukts bereitstellen lassen [71, S. 22].

188 Vgl. das Beispiel zum *Monitoring System* in Annex II [71, S. 41].

189 Vgl. das Beispiel zum *warming therapy device for premature babies* in Annex II [71, S. 41] oder zum *PACS* (Picture Archiving and Communication System) [71, S. 42].

190 So z. B. bei Magnetresonanzgeräten, Annex II [71, S. 42]. Selbst eine Manipulation der Internetverbindung begründet kein schwerwiegendes Vorkommnis, da Medizinprodukte autonom funktionieren sollen [71, S. 20 f.]; siehe das Beispiel zum *Mobile X‑ray*, [71, S. 43].

191 In vielen Fällen ist die Stärke der elektrischen Impulse eingeschränkt – z. B. aus Leistungsgründen oder um die Sicherheit der Patienten nicht zu gefährden. Das schränkt das Schadenspotenzial ein.

192 Vgl. Art. 95 Abs. 1 MPVO. Ggf. muss der Hersteller die Bereitstellung des Produkts auf dem Markt beschränken, bestimmten Anforderungen unterwerfen oder das Produkt vom Markt nehmen bzw. zurückrufen.

193 Zur Frage, inwiefern die Gesamtrechtsordnung diesen Gefahren durch andere Regulierungsregime, insbesondere durch das Datenschutzrecht, begegnet, siehe v. a. 2.2.1.b). Die allgemeinen Aufsichtspflichten sehen v. a. Stichproben-Kontrollen vor (Art. 93 Abs. 1 MPVO).

194 Vgl. Erw.gr. 44 ff. MPVO sowie https://ec.europa.eu/health/md_eudamed/overview_de.

195 Beschluss der Kommission vom 19. April 2010 über die Europäische Datenbank für Medizinprodukte (Eudamed), K(2010) 2363, ABl. L 102/45, Erw.gr. 2 und Art. 2.

196 Eine Liste der zuständigen Behörden findet sich unter https://ec.europa.eu/tools/eudamed/#/screen/competent-authorities.

197 https://ec.europa.eu/commission/presscorner/detail/de/IP_10_443.

198 Den Zeitpunkt der Inbetriebnahme des Registers gibt das Bundesministerium für Gesundheit durch eine Rechtsverordnung bekannt (§ 37 Nr. 1 Implantateregistergesetz [IRegG]).

199 Cochleaimplantate werden ebenfalls den Gehirn-Computer-Schnittstellen zugeordnet [1, S. 210 ff.].

200 Sonderanfertigungen oder Implantate mit Sonderzulassung (§ 15 Abs. 1 Nr. 1, § 2 Nr. 2, 3 IRegG) sind von der Registrierpflicht ausgenommen, da diese neben dem konkreten Einzelfall keine validen, interpretierbaren Daten liefern, vgl. BT-Drucks. 19/10523, S. 84 f.

201 Zu der datenschutzrechtlichen Gestaltung des Implantateregisters sowie zur Kritik siehe Bahner [209, S. 80 f., 83].

202 Ebenfalls bemängelt werden die fehlenden Sanktionen bei Verstoß gegen die Meldepflichten [209, S. 82].

203 Dazu auch 2.2.c.

204 V. a. § 8a des Gesetzes über das Bundesamt für Sicherheit in der Informationstechnik (BSIG) i. V. m. der Verordnung zur Bestimmung Kritischer Infrastrukturen (BSI-KritisV).

205 § 6 Abs. 1 Nr. 2 BSI-KritisV, siehe hierzu Tschammler [211, S. 511]. Hersteller von Gehirn-Computer-Schnittstellen bzw. Anbieter zusätzlicher Dienste gehören auch nicht zu den „Unternehmen im besonderen öffentlichen Interesse“ (§ 2 Abs. 14 BSIG). Kommt die Gehirn-Computer-Schnittstelle allerdings ausschließlich im klinischen Umfeld und stationär (z. B. bei schwerer Epilepsie) zum Einsatz, ist sie dagegen eine kritische Dienstleistung (§ 6 Abs. 1 Nr. 1 BSI-KritisV).

206 Etwas anderes gilt, wenn der App-Anbieter oder Hersteller eigene Sever betreibt und die Schwelle der Kleinst- und kleinen Unternehmen überschreitet (§ 8d Abs. 4 S. 1 BSIG).

207 Diese Anforderungen ergeben sich aus der *NIS-Richtlinie* (EU) 2016/1148 des Europäischen Parlaments und des Rates vom 6. Juli 2016 über Maßnahmen zur Gewährleistung eines hohen gemeinsamen Sicherheitsniveaus von Netz- und Informationssystemen in der Union. Zusätzlich treffen die Anbieter digitaler Dienste Meldepflichten (§ 8c Abs. 3 BSIG); das BSI kann Maßnahmen verlangen, um Sicherheitsmängel zu beseitigen (§ 8c Abs. 4 BSIG). Betreiber haben hierbei den Stand der Technik zu berücksichtigen (§ 8c Abs. 2 BSIG), nicht aber (wie Betreiber Kritischer Infrastrukturen, § 8a Abs. 1 S. 2 BSIG) einzuhalten.

208 Zu dem Projekt ManiMed, siehe Fn. 34.

209 Die MPVO ist im Verhältnis zum Produktsicherheitsrecht, das maßgeblich durch die Produktsicherheitsrichtlinie (2001/95/EG) geprägt ist, *Lex specialis*. Die EU-Kommission plant, die Produktsicherheitsrichtlinie zu überarbeiten, um u. a. die Cybersicherheit vernetzter Produkte zu verbessern [213, S. 3]. Allgemeine Anforderungen an die Cybersicherheit mit dem Internet verbundener Geräte ergeben sich mittlerweile auch aus der Funkanlagenrichtlinie (2014/53/EU) in Verbindung mit der delegierten Verordnung (EU) 2022/30 der Kommission vom 29. Oktober 2021 zur Ergänzung der Richtlinie 2014/53/EU des Europäischen Parlaments und des Rates im Hinblick auf die Anwendung der grundlegenden Anforderungen, auf die in Art. 3 Abs. 3 Buchstaben d, e und f der Richtlinie Bezug genommen wird.

210 Auch Implantate sind Produkte i. S. v. § 2 ProdHaftG [214, Rn. 30] – ebenso externe Gehirn-Computer-Schnittstellen eingebetteter Software [214, Rn. 21, 27].

211 Siehe hierzu bereits B.II.1.b) sowie das IT-Grundschutz-Kompendium unter SYS.4.4 [172].

212 Siehe z. B. die *Guidelines for Securing the Internet of Things* der Agentur der Europäischen Union für Cybersicherheit (ENISA).

213 Allerdings muss die Sicherheitslücke im Zeitpunkt des Inverkehrbringens bekannt gewesen sein [218, S. 1843 f.]. Nach Inverkehrbringen ist der Hersteller noch zur Marktbeobachtung verpflichtet [218, S. 1844]. Nur in Ausnahmefällen könnten die Marktüberwachungsbehörden die Bereitstellung eines Updates nach § 26 Abs. 2 S. 1 ProdSG anordnen [219, S. 628 f.].

214 Richtlinie (EU) 2019/771 des Europäischen Parlaments und des Rates vom 20. Mai 2019 über bestimmte vertragsrechtliche Aspekte des Warenkaufs, die für *Waren mit digitalen Elementen* gilt. Bei ihnen handelt es sich um bewegliche körperliche Gegenstände, die in einer Weise digitale Inhalte oder digitale Dienstleistungen enthalten oder mit ihnen verbunden sind, dass die Waren ihre Funktionen ohne diese digitalen Inhalte oder digitalen Dienstleistungen nicht erfüllen könnten (Art. 2 Nr. 5 lit. b).

215 Gesetz zur Regelung des Verkaufs von Sachen mit digitalen Elementen und anderer Aspekte des Kaufvertrags vom 25.06.2021, BGBl. 2021 Teil I Nr. 37, S. 2133 ff. Für digitale Inhalte oder Dienstleistungen ergibt sich die Aktualisierungspflicht aus § 327f BGB, vgl. Gesetz zur Umsetzung der Richtlinie über bestimmte vertragsrechtliche Aspekte der Bereitstellung digitaler Inhalte und digitaler Dienstleistungen vom 25.06.2021, BGBl. 2021 Teil I Nr. 37, S. 2123 ff.

216 Erw. gr. 48 der Richtlinie (EU) 2019/770 des Europäischen Parlaments und des Rates vom 20. Mai 2019 über bestimmte vertragsrechtliche Aspekte der Bereitstellung digitaler Inhalte und digitaler Dienstleistungen*.*

217 Vgl. auch den Gesetzentwurf der Bundesregierung für ein Gesetz zur Umsetzung der Richtlinie über bestimmte vertragsrechtliche Aspekte der Bereitstellung digitaler Inhalte und digitaler Dienstleistungen vom 13.01.2021, verfügbar unter https://www.bmjv.de/SharedDocs/Gesetzgebungsverfahren/Dokumente/RegE_BereitstellungdigitalerInhalte.pdf, S. 60.

218 So jedenfalls in Bezug auf Datenverarbeitungen der Regierungsentwurf vom 13.01.2021 (Fn. 218), S. 60.

219 *Europäische Kommission*, Vorschlag für eine Verordnung des Europäischen Parlaments und Rates zur Festlegung harmonisierter Vorschriften für Künstliche Intelligenz (Gesetz über Künstliche Intelligenz) und zur Änderung bestimmter Rechtsakte der Union vom 21.04.2021, 2021/0106(COD).

220 „Systeme der künstlichen Intelligenz“ definiert Art. 3 Nr. 1 als „Software, die mit einer oder mehreren der in Anhang I aufgeführten Techniken und Konzepte entwickelt worden ist und im Hinblick auf eine Reihe von Zielen, die vom Menschen festgelegt werden, Ergebnisse wie Inhalte, Vorhersagen, Empfehlungen oder Entscheidungen hervorbringen kann, die das Umfeld beeinflussen, mit dem sie interagieren“.

221 Siehe hierzu bereits Fn. 22. Anhang I KI-VO nennt u. a. „Konzepte des maschinellen Lernens“ (lit. a) und „statistische Ansätze“ (lit. b).

222 Vgl. zu Medizinprodukten Erw.gr. 30 KI-VO. Die Regulierung erstreckt sich auf den gesamten „New Legislative Framework“ der EU, vgl. Annex II.

223 Vgl. Art. 6 (1) KI-VO. Eine Sicherheitskomponente zeichnet sich dadurch aus, dass sie entweder eine Sicherheitsfunktion erfüllt oder bei Nicht- oder Fehlfunktion die Patientensicherheit gefährdet (vgl. Art. 3 (14) KI-VO).

224 Art. 8 ff., v. a. Art. 15 KI-VO*.* Entspricht ein in Verkehr gebrachtes KI-System nicht (mehr) den Anforderungen, muss dessen Anbieter dies korrigieren, Art. 21 KI-VO.

225 Art. 9, 17, 61 KI-VO.

226 Art. 15 Abs. 3 UAbs. 1 KI-VO.

227 Art. 12 sowie Art. 20 KI-VO*.* Der Entwurf enthält auch Anforderungen an die „Nutzer“ der Systeme (Art. 29). Sie müssen die Anweisungen zur Verwendung und Überwachung des Produkts beachten. Der persönliche, nicht professionelle Gebrauch schließt diese Nutzerpflichten aus, vgl. Art. 3 (4). „Nutzer“ kann aber ggf. (auch) das medizinisch-technische Personal sein, das die Gehirn-Computer-Schnittstelle einrichtet.

228 Zur Problematik des „Internet of Bodies“ (dt. Internet der Körper, da internetfähige Implantate menschliche Körper vernetzen) vgl. Matwyshyn [101, v. a. S. 116 ff.].

229 Siehe 2.2.2.a).

230 Maßgeblich wäre dann die Funktionsweise und die Interaktion des Produkts mit dem Körper des Nutzers – dies bilden die Risikogruppen der MPVO ohnehin ab [44, S. 79 f.]. Zudem beugt eine gemeinsame Regulierung Wertungskonflikten zwischen parallelen Regulierungsrahmen vor, die gleiche oder ähnliche Produkte unterschiedlich bewerten [196, S. 17]. Für eine Regulierung sog. *Cognitive Enhancement Devices* im Rahmen des Medizinprodukterechts sprechen sich Maslen et al. aus [44, 80 ff.]. Alternativ könnte die Europäische Kommission im Wege eines delegierten Rechtsakts gem. Art. 1 Abs. 5 MPVO die Liste des Anhang XVI erweitern.

231 Sicherheit und Leistung werden im Rahmen der klinischen Bewertung (Art. 2 Nr. 44 MPVO) untersucht, vgl. Art. 61 ff. MPVO.

232 Diese Anforderungen sollten für alle, auch bereits auf dem Markt befindliche Geräte gelten („Security Parity“) [101, S. 131 f.].

233 Die Koordinierungsgruppe Medizinprodukte hat u. a. zur Aufgabe, bei der Entwicklung von Leitlinien „für die wirksame und harmonisierte Durchführung [der MPVO und] Anwendung der grundlegenden Sicherheits- und Leistungsanforderungen“ *mitzuwirken* (Art. 105 lit. c MPVO).

234 Zur Bindungswirkung technischer Standards, siehe auch Yu [169, S. 167 ff.]. Vgl. für den US-amerikanischen Kontext Kersbergen [104, S. 416 f.].

235 Sekundäre Rechtsakte könnten verbindlich zur Verschlüsselung (und anderen Sicherheitsmaßnahmen) verpflichten, vgl. hierzu Fn. 177. Dabei sollte auch der Mindeststandard des Verschlüsselungsalgorithmus definiert werden [77, Rn. 34, 34d].

236 Neben dem deutschen BSI könnte auch die Agentur der Europäischen Union für Cybersicherheit (ENISA) mitwirken.

237 Bspw. kann dies mithilfe von Zertifikaten geschehen [98, S. 136 f.].

238 Siehe z. B. den „*BCI Anonymizer*“ von Howard Jay Chizeck und Tamara Bonaci: Patent US20140228701A1, https://patents.google.com/patent/US20140228701A1/en; vgl. hierzu Bonaci, Calo und Chizeck [14, S. 37 f.].

239 Ähnliches gilt auch für implantierbare Kardioverter-Defibrillatoren, die unangenehme, wenn auch nicht tödliche, elektrische Stromstöße abgeben können.

240 Die Liste der Nebenwirkungen von Neurostimulatoren ist lang: Neben Gedächtnisschwierigkeiten, Sehstörungen und psychischen Auswirkungen, wie Angstgefühlen, Halluzinationen oder Depressionen, können auch epileptischen Anfälle auftreten [45, S. 10].

241 Diese Kritik äußerte in Bezug auf die US-amerikanische Rechtslage auch Kersbergen [104, S. 412 ff.]: Angreifer hätten es bislang vor allem auf Patientendaten abgesehen; Angriffe auf die körperliche Integrität kämen selten bis gar nicht vor [104, S. 414].

242 Auch das Datenschutzrecht verpflichtet den Verantwortlichen zu Korrekturmaßnahmen, um die Datensicherheit (wieder-)herzustellen (Art. 32 Abs. 1 DSGVO). Siehe dazu B.II.1.b).

243 Bspw. beschloss die Europäische Kommission eine delegierte Verordnung zur Ergänzung der Funkanlagenrichtlinie, wodurch zahlreiche vernetzte Geräte IT-Sicherheitsanforderungen erfüllen müssen, vgl. die delegierte Verordnung (EU) 2022/30 (vgl. Fn. 209). Ferner könnten verpflichtend Kontaktstellen der Hersteller bei Vorfällen und ggf. Ansprechstellen der Branche zu benennen sein, ähnlich wie dies bereits bei Kritischen Infrastrukturen vorgesehen ist (§ 8b Abs. 3 und Abs. 5 BSIG).

244 Die Marktüberwachungsbehörden sind im Bereich der Produktsicherheit zuständig, siehe § 25 Abs. 1 ProdSG. Vgl. genauer die Verordnung (EU) 2019/1029 des Europäischen Parlaments und Europäischen Rates vom 20. Juni 2019 über Marktüberwachung und die Konformität von Produkten sowie zur Änderung der Richtlinie 2004/42/EG und der Verordnungen (EG) Nr. 765/2008 und (EU) Nr. 305/2011.

245 Eine solche Kooperation ist verfassungsrechtlich zulässig und verstößt nicht gegen das Verbot einer Mischverwaltung [228, Rn. 20]. Auch die reibungsfreie Kooperation der Datenschutzbehörden (insbesondere des Bundesbeauftragten für den Datenschutz und die Informationsfreiheit, BfDI) und des BSI ist von besonderer Wichtigkeit [229, S. 46].

246 https://www.nist.gov/programs-projects/national-vulnerability-database-nvd. Siehe zu Meldesystemen für Vorkommnisse auch Matwyshyn [101, S. 132]. Störungen Kritischer Infrastrukturen sind bereits dem BSI zu melden (§ 8b Abs. 4 BSIG); es könnte darüber hinaus zur zentralen Meldestelle in *allen* Angelegenheiten der Sicherheit in der Informationstechnik avancieren (vgl. § 8b Abs. 1 BSIG, der diese Funktion auf Kritische Infrastrukturen begrenzt). Das BSI unterliegt der Aufsicht des Bundesministeriums des Innern und für Heimat, Stadtentwicklung und Bauwesen. Das kann Interessenkonflikte und Risiken verursachen, wenn es Sicherheitslücken für Zwecke der Strafverfolgung offen lässt [230, S. 231 ff.].

247 Dies ist bereits in §§ 5a, 7b, 7c 8b Abs. 6, 8c Abs. 4 BSIG bei Betreibern Kritischer Infrastrukturen, Anbietern digitaler Dienste etc. bzw. bei schwerwiegenden Vorkommnissen, die auf Sicherheitslücken der IT-Sicherheit beruhen (§ 85 Abs. 5 Nr. 1 MPDG), vorgesehen.

248 Einen Lügendetektor zu verwenden, ruft zahlreiche rechtliche Zulässigkeitsfragen auf den Plan [87, S. 3 ff.; 231, S. 433 ff.]. Zur US-amerikanischen Rechtslage, siehe Farahany [84, S. 351 ff.].

249 In den USA wurde ein Täter mithilfe der Herzfrequenzdaten seines Herzschrittmachers überführt, siehe hierzu Fn. 55. Eine parallele Situation entsteht bei Smart-Home-Geräten und Sprachassistenten: Auch sie speichern private, teils sehr intime, Informationen, die sich mitunter als Beweisquelle für Strafverfolgungsbehörde eignen [232, 233].

250 Zur Quellen-Telekommunikationsüberwachung und den damit verbundenen verfassungsrechtlichen Fragen siehe Martini und Fröhlingsdorf [124, S. 3 ff.].

251 Dies hat sich bislang v. a. im Zivilrecht gezeigt. Es hielt kaum Pflichten zur Gewährleistung der IT-Sicherheit und zur Bereitstellung von Aktualisierungen vor [156, S. 3346 f.; 234; 235].

252 Vgl. https://cyberpunk.fandom.com/wiki/Trauma_Team_International#2077.
